# Interaction and assembly of the DNA replication core proteins of Kaposi’s sarcoma-associated herpesvirus

**DOI:** 10.1128/spectrum.02254-23

**Published:** 2023-10-24

**Authors:** Lee-Wen Chen, Shie-Shan Wang, Li-Yu Chen, Hsiao-Yun Huang, Si-min He, Chien-Hui Hung, Chun-Liang Lin, Pey-Jium Chang

**Affiliations:** 1 Department of Respiratory Care, Chang Gung University of Science and Technology, Chiayi, Taiwan; 2 Department of Pediatric Surgery, Chang Gung Memorial Hospital, Chiayi, Taiwan; 3 School of Medicine, Chang Gung University, Taoyuan, Taiwan; 4 Graduate Institute of Clinical Medical Sciences, College of Medicine, Chang Gung University, Taoyuan, Taiwan; 5 Department of Nephrology, Chang Gung Memorial Hospital, Chiayi, Taiwan; Barnard College, Columbia University, New York, New York, USA

**Keywords:** KSHV, DNA replication complex, primase, helicase, DNA polymerase, Hsp90, protein-protein interaction

## Abstract

Kaposi’s sarcoma-associated herpesvirus (KSHV) encodes six highly conserved core replication proteins essential for the viral lytic DNA synthesis, including ORF6 (single-stranded DNA-binding protein), ORF9 (DNA polymerase), ORF40/41 (primase-associated factor), ORF44 (helicase), ORF56 (primase), and ORF59 (polymerase processivity factor). Since the protein-protein interactions among KSHV core replication proteins are largely unknown, this study aimed to decipher their interrelationships. Herein, we propose a protein-protein interaction network of these six core replication proteins according to the results obtained from confocal fluorescence microscopy, coimmunoprecipitation, and mammalian two-hybrid (GAL4/VP16) reporter assays. In this interaction network, ORF40/41 plays a central role in the connection of different replication subcomplexes. In addition to the well-conserved helicase-primase subcomplex (consisting of ORF44, ORF56, and ORF40/41) and the replisome subcomplex (consisting of ORF9 and ORF59), our data also suggest that several discrete, stable subcomplexes exist in the cell nucleus. Among these replication subcomplexes in the nucleus, the tetrameric subcomplex composed of ORF44, ORF56, ORF40/41, and ORF6 exhibited the ability to trigger a DNA damage response. By using an established GAL4/VP16-based reporter system and confocal fluorescence microscopy, we additionally found that the heat shock protein 90 (Hsp90) inhibitor, radicicol, significantly inhibited the formation of both the helicase-primase subcomplex and the replisome subcomplex in a dose-dependent manner. Collectively, these data not only provide further insights into the interaction and assembly of KSHV-encoded core replication proteins but also suggest a critical role of Hsp90 in assisting the construction of the viral core replication machinery.

**IMPORTANCE:**

Eukaryotic DNA replication is a highly regulated process that requires multiple replication enzymes assembled onto DNA replication origins. Due to the complexity of the cell’s DNA replication machinery, most of what we know about cellular DNA replication has come from the study of viral systems. Herein, we focus our study on the assembly of the Kaposi’s sarcoma-associated herpesvirus core replication complex and propose a pairwise protein-protein interaction network of six highly conserved viral core replication proteins. A detailed understanding of the interaction and assembly of the viral core replication proteins may provide opportunities to develop new strategies against viral propagation.

## INTRODUCTION

The lytic DNA replication of herpesviruses is an important model system to study eukaryotic DNA replication (1, 2). To ensure the correct assembly of a functional DNA replication complex, herpesviruses may have evolved multiple elaborate mechanisms that control the expression and interaction of different core replication proteins and prevent the mislocalization and accumulation of immature replication subcomplexes in the nucleus of host cells. Furthermore, due to the fact that most of anti-herpesvirus drugs are known to target viral replication enzymes (3 – 6), understanding the assembly of herpesviral core replication components in detail may be helpful to develop novel therapies against viral replication.

Based on current knowledge, all herpesviruses may use very similar DNA replication machineries to amplify their genomes (2, 7, 8). Herpes simplex virus type 1 (HSV-1), the prototype of the alpha herpesvirus subfamily, is a well-characterized member of human viruses. Previous studies have demonstrated that there are seven virus-encoded replication proteins essential for viral DNA synthesis (1, 2, 9). These viral proteins are the origin binding protein UL9 and six conserved core replication proteins, namely, UL5 (helicase), UL8 (primase-associated factor), UL52 (primase), UL30 (DNA polymerase), UL42 (polymerase processivity factor), and ICP8 (single-stranded DNA-binding protein). Among them, UL5, UL8, and UL52 can form a heterotrimeric helicase-primase subcomplex, whereas UL30 and UL42 form a heterodimeric replisome subcomplex (1, 2). For the assembly of a functional replication complex, evidence from earlier studies revealed that the helicase-primase subcomplex component UL8 was capable of interacting with the DNA polymerase component UL30 (10). In addition, UL8 and UL52 in the helicase-primase subcomplex have been reported to either functionally or physically interact with ICP8 (9, 11 – 14). Similarly, Epstein-Barr virus (EBV), another widespread human virus belonging to the gamma-1 herpesvirus subfamily, also encodes seven essential DNA replication proteins (8, 15 – 17). These viral proteins are the origin binding protein (BZLF1 or Zta), the single-stranded DNA-binding protein (BALF2), the helicase-primase subcomplex (BBLF4, BSLF1, and BBLF2/3), and the replisome subcomplex (BALF5 and BMRF1). Besides the interactions of the components within the conserved helicase-primase and replisome subcomplexes, the DNA polymerase component BALF5 could also interact with each component of the BBLF4–BSLF1–BBLF2/3 subcomplex (8, 18). Despite the fact that both HSV-1 and EBV have been extensively studied for several decades, some details of the protein-protein interactions involved in the assembly of their DNA replication machineries are still not fully understood.

Kaposi’s sarcoma-associated herpesvirus (KSHV), also known as human herpesvirus 8), is the most recently identified human herpesvirus belonging to the gamma-2 herpesvirus subfamily (19, 20). This virus is etiologically implicated in the development of Kaposi’s sarcoma, primary effusion lymphomas (PELs), and multicentric Castleman’s disease (21). For the viral lytic DNA replication, eight viral proteins including two origin-binding proteins (ORF50 and K8) and six conserved core replication proteins are required (21 – 24). The six essential core replication proteins are ORF6 (single-stranded DNA-binding protein, SSB), ORF9 (DNA polymerase, POL), ORF40/41 (primase-associated factor, PAF), ORF44 (helicase, HEL), ORF56 (primase, PRI), and ORF59 (polymerase processivity factor, PPF). Similar to the homologs of HSV-1 and EBV, the KSHV ORF9 is known to form heterodimers with ORF59 (25 – 27). However, it is currently unclear whether the ORF44, ORF56, and ORF40/41 proteins, like their homologs of HSV-1 and EBV, form a stable helicase-primase subcomplex in cells. In comparison with HSV-1 and EBV, very few studies have focused on the interaction and assembly of the essential core replication proteins of KSHV.

In this study, we aimed to investigate the protein-protein interactions of KSHV-encoded core replication proteins and the assembly of their associated replication intermediates. Different methods including confocal fluorescence microscopy, coimmunoprecipitation, and mammalian two-hybrid (GAL4/VP16) reporter assays were utilized in the study. Based on the results obtained, a protein-protein interaction network of the six viral core replication proteins was proposed. Importantly, we found that radicicol, a heat shock protein 90 (Hsp90) inhibitor, could disrupt the formation of both the conserved helicase-primase subcomplex (consisting of ORF44, ORF56, and ORF40/41) and the replisome subcomplex (consisting of ORF9 and ORF59). Our results, therefore, suggest that Hsp90 may play a critical role in the assembly of KSHV core replication complex.

## RESULTS

### Subcellular localization of six KSHV-encoded core replication proteins

**Fig 1:**
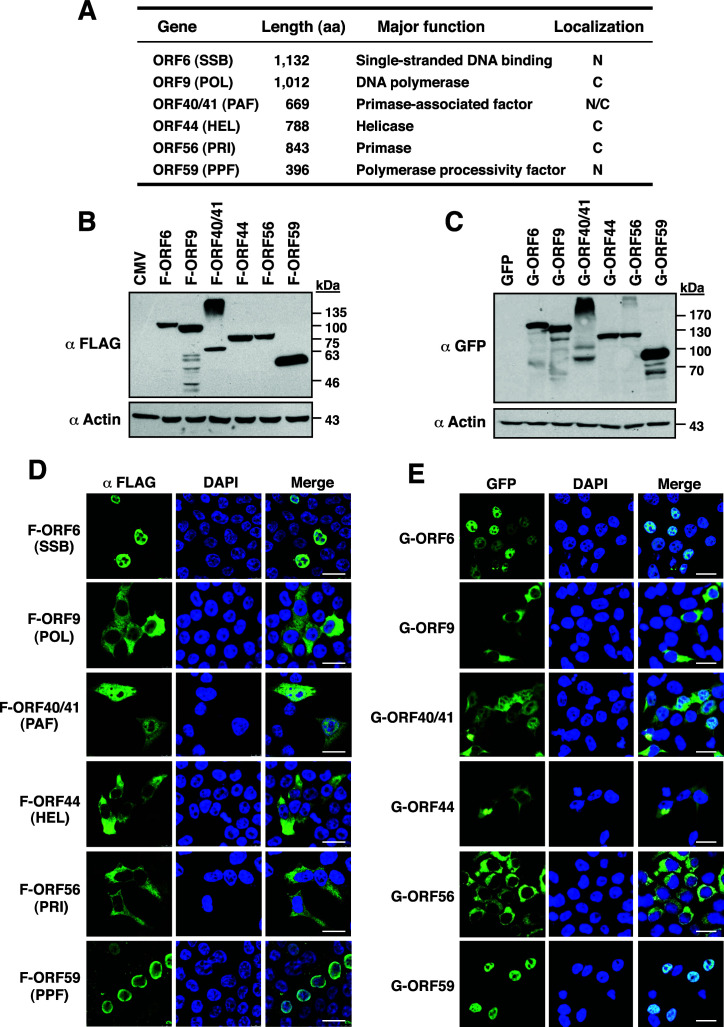
Expression and subcellular localization of KSHV-encoded core replication proteins in 293T cells. (**A**) Summary of the functional properties and the subcellular localization of the six viral core replication proteins. (**B and C**) Western blot analysis of the expression of FLAG-tagged or GFP-tagged core replication proteins in 293T cells. The experiments were performed twice independently with similar results. (**D and E**) Confocal microscopic analysis of the subcellular localization of FLAG-tagged or GFP-tagged core replication proteins in 293T cells. Scale bars, 20 µM. The experiments were repeated at least three times independently with similar results.

To characterize the expression and subcellular localization of KSHV core replication proteins, the viral DNA fragments corresponding to the coding regions of ORF6 (SSB), ORF9 (POL), ORF40/41 (PAF), ORF44 (HEL), ORF56 (PRI), and ORF59 (PPF) (Fig. 1A) were individually cloned into the expression vector pFLAG-CMV-2 (carrying a coding sequence of a FLAG tag) or pEGFP-C2 [carrying a coding sequence of a green fluorescent protein (GFP) tag]. The resultant plasmids were transfected into 293T cells, and the expressions of these core replication proteins in cells were analyzed by Western blotting. Our results showed that the molecular masses of F-ORF6, F-ORF9, F-ORF40/41, F-ORF44, F-ORF56, and F-ORF59 were 120, 110, 70, 80, 85, and 55 kDa, respectively (Fig. 1B), whereas the molecular masses of GFP-ORF6, GFP-ORF9, GFP-ORF40/41, GFP-ORF44, GFP-ORF56, and GFP-ORF59 were 150, 140, 100, 110, 115, and 85 kDa, respectively (Fig. 1C). Although all viral proteins migrated to their predicted positions in gels, we noticed that ORF40/41 with a FLAG tag or GFP tag was prone to form high-molecular-mass aggregates in the experiments (Fig. 1B and C). The subcellular localization of these core replication proteins in 293T cells was examined by confocal fluorescence microscopy. Confocal microscopic analysis revealed that F-ORF6 and F-ORF59 were mainly localized in the nucleus, whereas F-ORF9, F-ORF44, and F-ORF56 were restricted to the cytoplasm (Fig. 1D). Only F-ORF40/41 was shown in both the nucleus and cytoplasm (Fig. 1D). Consistently, the viral core replication proteins with a GFP tag also exhibited the same subcellular localizations to their respective counterparts with a FLAG tag in 293T cells (Fig. 1E). Notably, three core replication proteins with enzymatic activities, including ORF9, ORF44, and ORF56, were confined to the cytoplasm, suggesting that the cytoplasmic retention of these core replication proteins may be an important regulatory point to prevent untimely DNA synthesis.

### Dynamic changes in the subcellular localization of ORF56 in the presence of other core replication proteins

**Fig 2:**
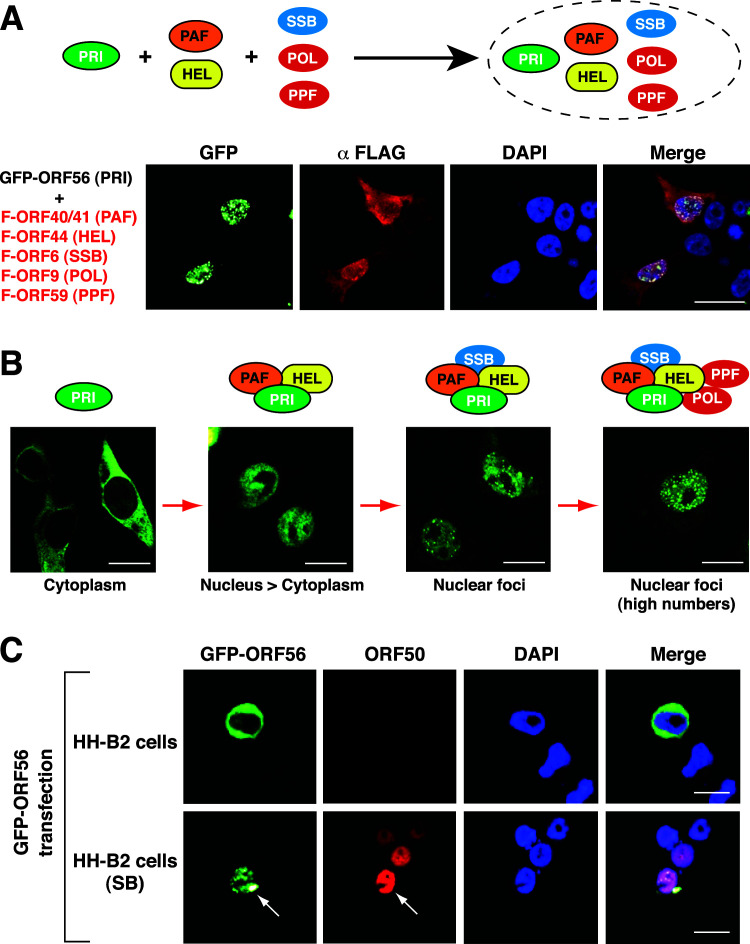
Changes in the subcellular localization of GFP-ORF56 induced by other viral core replication proteins. (**A**) Confocal microscopic analysis of the subcellular localization of GFP-ORF56 in the presence of five FLAG-tagged core replication proteins in 293T cells. Scale bars, 20 µM. (**B**) A proposed model for changes in the GFP-ORF56 subcellular localization during the assembly of the viral core replication complex. When specific sets of core replication proteins were coexpressed, different localization patterns of GFP-ORF56 were observed in cells, including (i) exclusively localized in the cytoplasm, (ii) mainly localized in the nucleus, and (iii) mainly localized in the nucleus with puncta formation. Scale bars, 10 µM. (**C**) Subcellular localization of GFP-ORF56 in HH-B2 cells untreated or treated with SB. In the experiments, HH-B2 cells transfected with pCMV-GFP-ORF56 were left untreated or treated with SB for 24 h, and then the subcellular localization of GFP-ORF56 in transfected cells was analyzed by confocal microscopy. Scale bars, 10 µM. Notably, SB treatment induced the formation of GFP-ORF56 puncta in the cell nucleus (white arrow) and the expression of the viral lytic protein ORF50 (red). All experiments shown above were performed at least three times independently with similar results.

ORF56 is a primase protein essential for initiating the viral DNA synthesis. As shown in Fig. 1, GFP-ORF56 was mainly localized in the cytoplasm when expressed individually. However, when GFP-ORF56 was coexpressed with the other five core replication proteins including F- ORF40/41, F-ORF44, F-ORF6, F-ORF9, and F-ORF59 in 293T cells, GFP-ORF56 displayed a punctate pattern in the nucleus (Fig. 2A). To examine the subcellular localization changes of GFP-ORF56 in more detail, different combinations of the viral core replication proteins were coexpressed with GPF-ORF56 in 293T cells. Confocal microscopic analysis found that coexpression of both F-ORF40/41 and F-ORF44 with GFP-ORF56 allowed the nuclear transport of most GFP-ORF56 (Fig. 2B; Fig. S1, panel a). However, coexpression of F-ORF9, F-ORF59, and/or F-ORF6 with GFP-ORF56 did not convey GFP-ORF56 from the cytoplasm to the nucleus (Fig. S1, panels b and c). Furthermore, omission of either F-ORF40/41 or F-ORF44 failed to mediate the nuclear transport of GFP-ORF56, even in the presence of other core replication proteins in transfected cells (Fig. S1, panels d–g). These results suggested that both ORF40/41 and ORF44 were absolutely required for the nuclear transport of ORF56, thereby forming a trimeric helicase-primase subcomplex.

Although coexpression of both F-ORF40/41 and F-ORF44 was essential for the nuclear transport of GFP-ORF56, there were no punctate structures detected under the condition (Fig. 2B). To further investigate the minimal requirement for the formation of GFP-ORF56 puncta in the nucleus, different core replication proteins were coexpressed with the helicase-primase subcomplex consisting of GFP-ORF56, F-ORF40/41, and F-ORF44 in 293T cells (Fig. S2). We found that the formation of GFP-ORF56 puncta in the nucleus required the participation of F-ORF6 but not F-ORF9 and/or F-ORF59 (Fig. 2B; Fig. S2). As compared to GFP-ORF56 puncta formed by coexpression of GFP-ORF56 with F-ORF40/41, F-ORF44, and F-ORF6, a full set of the viral core replication proteins coexpressed in cells substantially increased numbers of GFP-ORF56 puncta in the nucleus (Fig. 2B). In KSHV-positive HH-B2 cells transfected with the plasmid encoding GFP-ORF56, we also found that GFP-ORF56 was expressed exclusively in the cytoplasm under the latent condition (Fig. 2C). However, GFP-ORF56 entered the nucleus and formed a punctate pattern in HH-B2 cells after treatment with sodium butyrate (SB), a lytic-inducing agent (Fig. 2C). Overall, these data suggest that the subcellular localization of ORF56 is tightly controlled by other viral core replication proteins.

### Colocalization of GFP-ORF56 puncta with the DNA damage-associated foci in the cell nucleus

**Fig 3:**
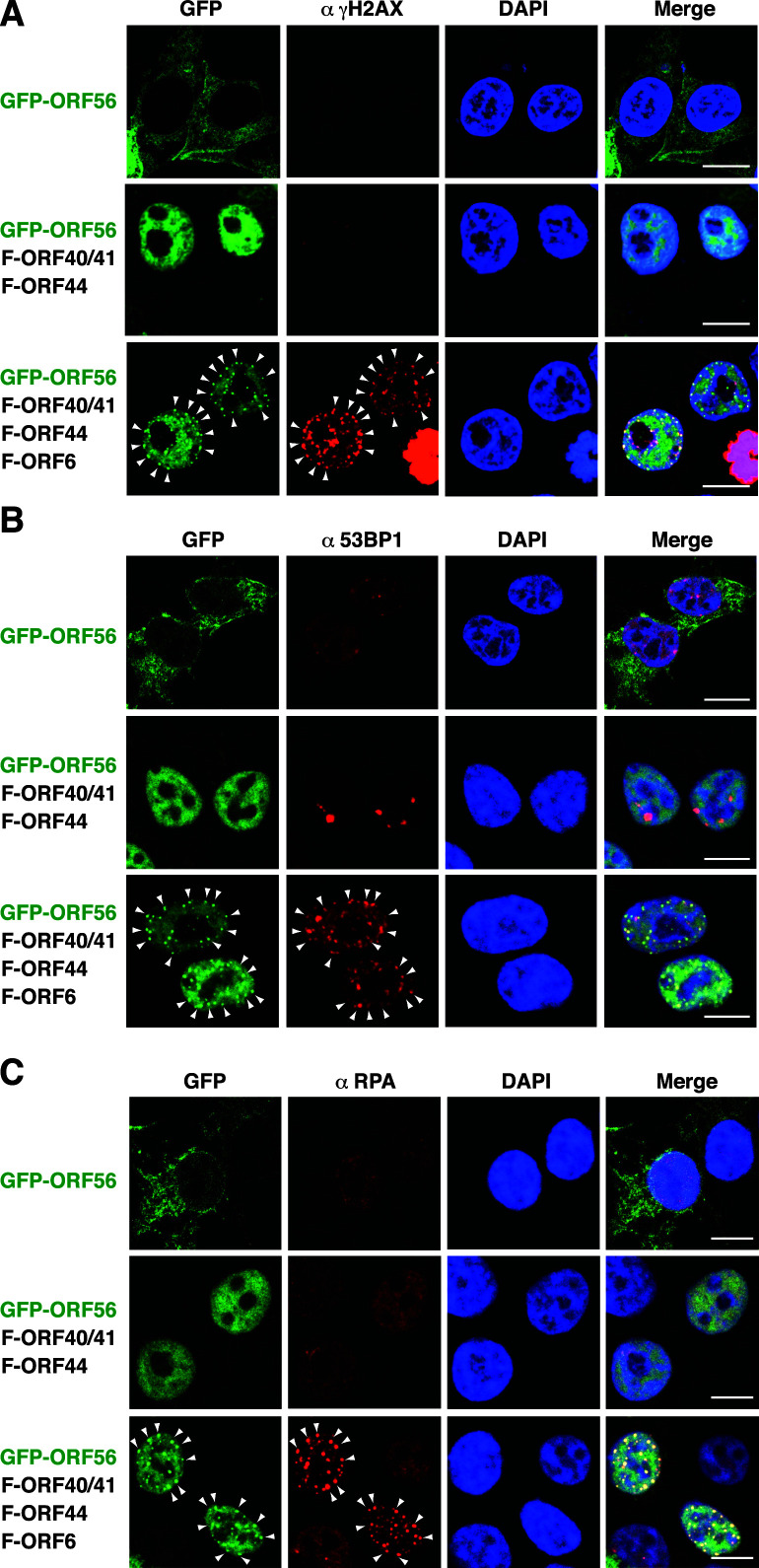
Colocalization of GFP-ORF56 puncta with the focal sites of γH2AX, 53BP1, and RPA in the cell nucleus. After 293T cells were transfected with the indicated expression plasmids for 24 h, the transfected cells were analyzed for the subcellular localization of GFP-ORF56 and the DNA damage markers including γH2AX (**A**), 53BP1 (**B**), and RPA (**C**) by confocal fluorescence microscopy. Scale bars, 10 µM. As noted, the foci formation ofγH2AX, 53BP1, or RPA was mainly detected in 293T cells expressing GFP-ORF56, F-ORF40/41, F-ORF44, and F-ORF6 together. White arrows indicate the colocalization of GFP-ORF56 puncta with γH2AX, 53BP1, or RPA foci in the nucleus of transfected cells. All experiments were performed at least twice independently with similar results.

Since GFP-ORF56 could form nuclear puncta in the presence of F-ORF44, F-ORF40/41, and F-ORF6, we examined whether GFP-ORF56 puncta were associated with cellular DNA damage. Accordingly, several sensitive DNA damage markers such as phosphorylated histone H2AX (γH2AX) (28, 29), p53-binding protein 1 (53BP1) (30 – 32), and replication protein A (RPA) (33 – 35) were examined in these transfected cells. As compared to 293T cells transfected with pCMV-GFP-ORF56 alone, 293T cells cotransfected with three expression plasmids including pCMV-GFP-ORF56, pCMV-F-ORF40/41, and pCMV-F-ORF44 did not significantly induce the foci formation ofγH2AX, 53BP1, or RPA (Fig. 3A through C). However, 293T cells cotransfected with four expression plasmids including pCMV-GFP-ORF56, pCMV-F-ORF40/41, pCMV-F-ORF44, and pCMV-F-ORF6 substantially triggered the formation of γH2AX, 53BP1, and RPA foci (Fig. 3A through C). Importantly, we found that most GFP-ORF56 puncta could colocalize with the focal sites ofγH2AX, 53BP1, or RPA in the cell nucleus (Fig. 3A through C), suggesting that the formation of the tetrameric ORF56–ORF44–ORF40/41–ORF6 subcomplex in the nucleus may cause cellular DNA damage.

### Confocal microscopic analysis of the pairwise protein-protein interactions among the viral core replication proteins

**Fig 4:**
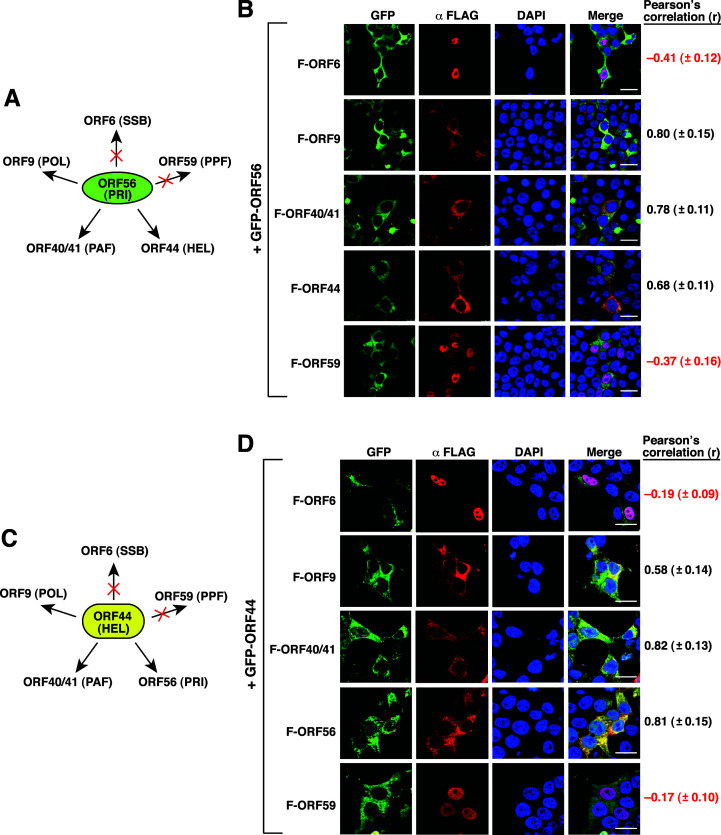
Analysis of the colocalization of GFP-ORF56 or GFP-ORF44 with the other KSHV-encoded core replication proteins in 293T cells. (**A**) Summary of the colocalization of GFP-ORF56 with the other five core replication proteins in 293T cells. “×,” no colocalization. (**B**) Quantitative analysis of the colocalization between GFP-ORF56 and the indicated FLAG-tagged core replication proteins in 293T cells. The degree of colocalization of two different fluorescent labels in cells was determined by the Pearson’s correlation coefficient (*r*). Scale bars, 20 µM. Data are expressed as means ± SD (*n* = 15 cells, three independent experiments). (**C**) Summary of the colocalization of GFP-ORF44 with the other five core replication proteins in 293T cells. “×,” no colocalization. (**D**) Quantitative analysis of the colocalization between GFP-ORF44 and the indicated FLAG-tagged core replication proteins in 293T cells. The degree of colocalization of two different fluorescent labels in cells was determined by the Pearson’s correlation coefficient (*r*). Scale bars, 20 µM. Data are expressed as means ± SD (*n* = 15 cells, three independent experiments).

Although the viral proteins ORF56, ORF40/41, and ORF44 were believed to form a conserved helicase-primase subcomplex in the cell, the protein-protein interactions among these three proteins actually remain unclear. Moreover, little is known about the interaction of the helicase-primase subcomplex components with the other core replication proteins. To determine the pairwise protein-protein interactions among these viral replication proteins, we first chose GFP-ORF56 as a target protein to analyze its association with the other FLAG-tagged core replication proteins in 293T cells by confocal fluorescence microscopy (Fig. 4A and B). Confocal microscopic analysis showed that GFP-ORF56 had a high degree of colocalization with F-ORF9, F-ORF40/41, and F-ORF44 (Fig. 4A and B; Pearson’s correlation coefficient *r* = 0.80 ± 0.15, *r* = 0.78 ± 0.11, and *r* = 0.68 ± 0.11, respectively). However, no significant colocalization was detected between GFP-ORF56 and F-ORF6 or F-ORF59 (Fig. 4A and B; *r* = –0.41 ± 0.12 and *r* = –0.37 ± 0.16, respectively). In these experiments, we additionally noticed that coexpression of GFP-ORF56 and F-ORF40/41 in cells caused retention of F-ORF40/41 in the cytoplasm.

Next, different GFP-tagged core replication proteins were separately used as a target protein to characterize its interactions with other core replication proteins. When GFP-ORF44 was coexpressed with different FLAG-tagged core replication proteins in 293T cells, we found that GFP-ORF44 significantly colocalized with F-ORF9, F-ORF40/41, and F-ORF56 (*r* = 0.58 ± 0.14, *r* = 0.82 ± 0.13, and *r* = 0.81 ± 0.15, respectively) but not with F-ORF6 and F-ORF59 (*r* = –0.19 ± 0.09 and *r* = –0.17 ± 0.10, respectively) (Fig. 4C and D). Our results also showed that GFP-ORF44 could alter the subcellular localization of F-ORF40/41 from the nuclear-cytoplasmic distribution to the cytoplasmic retention (Fig. 4D).

**Fig 5:**
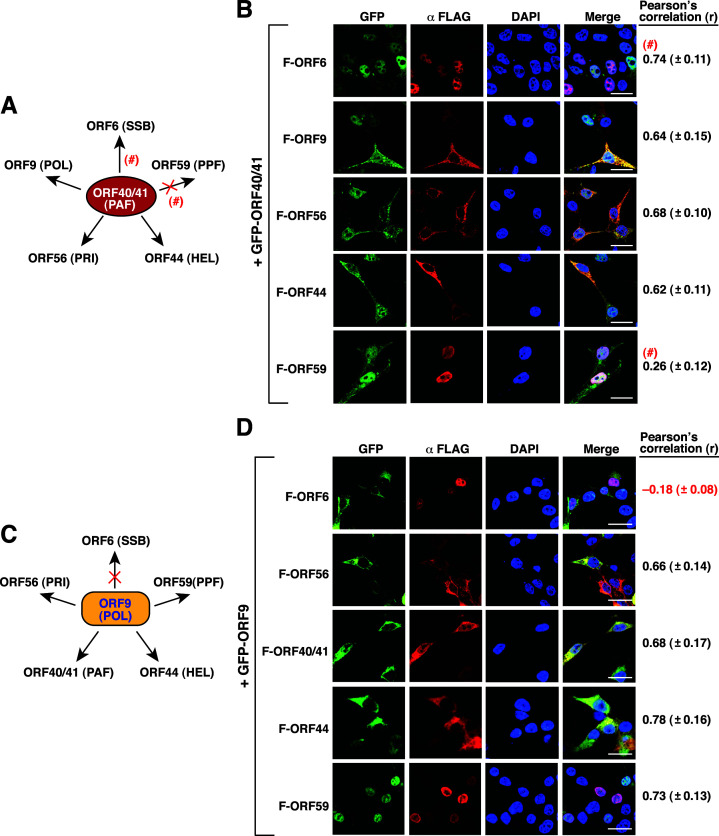
Analysis of the colocalization of GFP-ORF40/41 or GFP-ORF9 with the other KSHV-encoded core replication proteins in 293T cells. (**A**) Summary of the colocalization of GFP-ORF40/41 with the other five core replication proteins in 293T cells. “×,” no colocalization; “#,” additional validation required. (**B**) Quantitative analysis of the colocalization between GFP-ORF40/41 and the indicated FLAG-tagged core replication proteins in 293T cells. The colocalization between GFP-ORF40/41 and the indicated FLAG-tagged core replication proteins in cells was assessed by the Pearson’s correlation coefficient (*r*). Scale bars, 20 µM. Data are presented as means ± SD (*n* = 15 cells, three independent experiments). (**C**) Summary of the colocalization of GFP-ORF9 with the other five core replication proteins in 293T cells. “×,” no colocalization. (**D**) Quantitative analysis of the colocalization between GFP-ORF44 and the indicated FLAG-tagged core replication proteins in 293T cells. The colocalization between GFP-ORF44 and the indicated FLAG-tagged core replication proteins in cells was assessed by the Pearson’s correlation coefficient (*r*). Scale bars, 20 µM. Data are presented as means ± SD (*n* = 15 cells, three independent experiments).

When GFP-ORF40/41 served as a target protein, we observed that GFP-ORF40/41 seemed to colocalize with all five core replication proteins in 293T cells (*r* = 0.74 ± 0.11 for F-ORF6; *r* = 0.64 ± 0.15 for F-ORF9; *r* = 0.68 ± 0.10 for F-ORF56; *r* = 0.62 ± 0.11 for F-ORF44; *r* = 0.26 ± 0.12 for F-ORF59) (Fig. 5A and B). However, the value of Pearson’s correlation coefficient for the relationship between GFP-ORF40/41 and F-ORF59 was relatively low (*r* < 0.5). Since the fluorescence signals of GFP-ORF40/41 were diffusely distributed in both the nucleus and the cytoplasm (Fig. 5B), the association between GFP-ORF40/41 and different core replication proteins, particularly F-ORF59, needed to be further determined by using other approaches or methods (see below).

When GFP-ORF9 served as a target protein, GFP-ORF9 was shown to colocalize with the proteins including F-ORF56, F-ORF40/41, F-ORF44, and F-ORF59 (*r* = 0.66 ± 0.14, *r* = 0.68 ± 0.17, *r* = 0.78 ± 0.16, and *r* = 0.73 ± 0.13, respectively) but not with F-ORF6 (*r* = –0.18 ± 0.08) (Fig. 5C and D). Based on the image analysis of confocal fluorescence microscopy, we additionally found that (i) GFP-ORF9 could restrain F-ORF40/41 from going to the nucleus and (ii) GFP-ORF9 could be conveyed to the nucleus by F-ORF59 (Fig. 5D).

**Fig 6:**
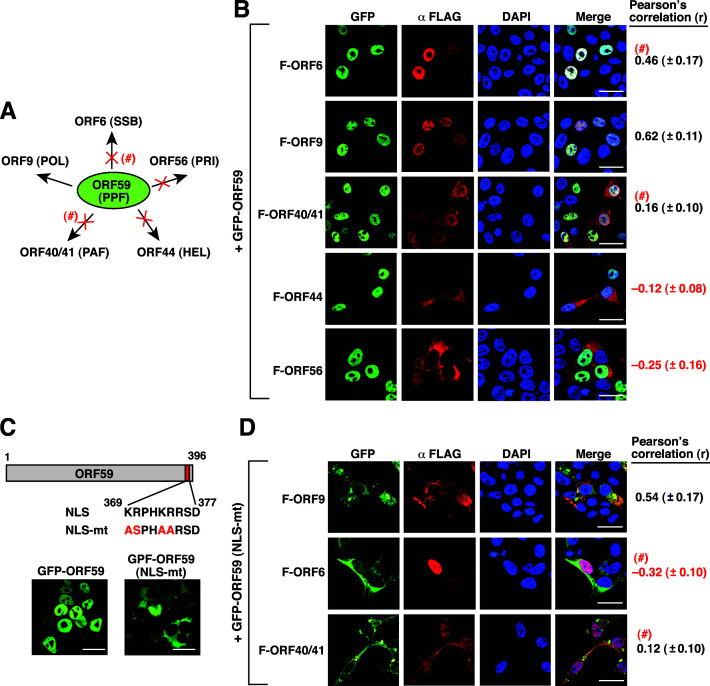
Confocal microscopic analysis of the colocalization between GFP-ORF59 and the other five core replication proteins in 293T cells. (**A**) Summary of the colocalization of GFP-ORF59 with the other five core replication proteins. “×,” no colocalization; “#,” additional validation required. (**B**) Quantitative analysis of the colocalization of GFP-ORF59 with the indicated FLAG-tagged core replication proteins in 293T cells. The degree of colocalization of GFP-ORF59 with the indicated FLAG-tagged core replication proteins in cells was determined by the Pearson’s correlation coefficient (*r*). Scale bars, 20 µM. Data are presented as means ± SD (*n* = 15 cells, three independent experiments). (**C**) Confocal microscopic analysis of the wild-type and NLS-mutated GFP-ORF59 constructs expressed in 293T cells. Scale bars, 20 µM. (**D**) Quantitative analysis of the colocalization between the NLS-mutated GFP-ORF59 and the indicated proteins including F-ORF9, F-ORF6, and F-ORF40/41. Data are presented as means ± SD (*n* = 15 cells, three independent experiments). Scale bars, 20 µM.

As shown in Fig. 1D and E, ORF59 is a nuclear protein. When GFP-ORF59 was coexpressed with other core replication proteins in 293T cells, we found that GFP-ORF59 showed little colocalization with F-ORF40/41, F-ORF44, and F-ORF56 (Fig. 6A and B; *r* = 0.16 ± 0.11, *r* = –0.12 ± 0.08, and *r* = –0.25 ± 0.16, respectively). This was consistent with the observational results shown in Fig. 4B (a negative association between GFP-ORF56 and F-ORF59), Fig. 4D (a negative association between GFP-ORF44 and F-ORF59), and Fig. 5B (a weak association between GFP-ORF40/41 and F-ORF59). Based on the degree of colocalization, only two proteins including F-ORF6 and F-ORF9 were the potential interacting partners of GFP-ORF59 (Fig. 6A and B; *r* = 0.46 ± 0.17 and *r* = 0.62 ± 0.11, respectively). Since GFP-ORF59 enabled the nuclear transport of F-ORF9 from the cytoplasm (Fig. 6B), the interaction between GFP-ORF59 and F-ORF9 was confirmed. Due to a moderate positive relationship between GFP-ORF59 and F-ORF6 (*r* < 0.5), the interaction between these two proteins needed to be further determined. To clarify this ambiguous protein-protein interactions, a GFP-ORF59 mutant defective in the nuclear transport was generated (Fig. 6C, NLS-mt). The GFP-ORF59(NLS-mt) construct contains point mutations within the nuclear localization signal (NLS) located between residues 369 and 377 (26). In the coexpression experiments, GFP-ORF59(NLS-mt) still showed a high degree of colocalization with F-ORF9 (Fig. 6D; *r* = 0.54 ± 0.17) although both proteins were retained in the cytoplasm. However, GFP-ORF59(NLS-mt) displayed little colocalization with F-ORF6 and F-ORF40/41 (Fig. 6D; *r* = –0.32 ± 0.10 and *r* = 0.12 ± 0.10, respectively). These results suggested that ORF9 might be the only core replication protein that interacts with ORF59 (Fig. 6A).

**Fig 7:**
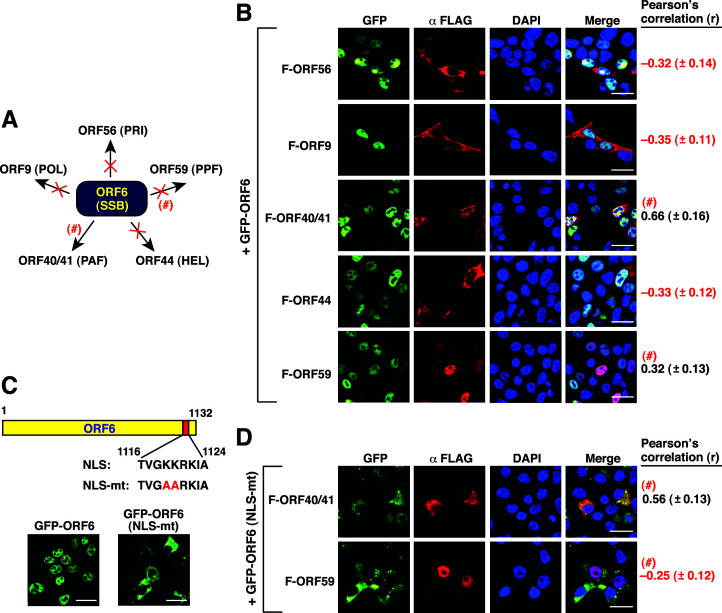
Confocal microscopic analysis of the colocalization between GFP-ORF6 and the other five core replication proteins in 293T cells. (**A**) Summary of the colocalization of GFP-ORF6 with the other five core replication proteins. “×,” no colocalization; “#,” additional validation required. (**B**) Quantitative analysis of the colocalization of GFP-ORF6 with the indicated FLAG-tagged core replication proteins in 293T cells. The degree of colocalization of GFP-ORF6 with the indicated FLAG-tagged core replication proteins in cells was determined by the Pearson’s correlation coefficient (*r*). Scale bars, 20 µM. Data are presented as means ± SD (*n* = 15 cells, three independent experiments). (**C**) Confocal microscopic analysis of the wild-type and NLS-mutated GFP-ORF6 constructs expressed in 293T cells. Scale bars, 20 µM. (**D**) Quantitative analysis of the colocalization between the NLS-mutated GFP-ORF6 and the indicated proteins including F-ORF40/41 and F-ORF59. Data are presented as means ± SD (*n* = 15 cells, three independent experiments). Scale bars, 20 µM.

Lastly, when GFP-ORF6 was coexpressed with other core replication proteins in 293T cells, our results found that a high degree of colocalization was detected between GFP-ORF6 and F-ORF40/41 (Fig. 7A and B; *r* = 0.66 ± 0.16). However, the core replication proteins including F-ORF56, F-ORF9, and F-ORF44 did not show any significant colocalization with GFP-ORF6 (Fig. 7A and B; *r* = –0.32 ± 0.14, *r* = –0.35 ± 0.11, and *r* = –0.33 ± 0.12, respectively). Additionally, a moderate degree of colocalization between GFP-ORF6 and F-ORF59 was detected in the experiment (Fig. 7B; *r* = 0.32 ± 0.13). To further clarify the association between GFP-ORF6 and F-ORF40/41 or F-ORF59, a NLS-mutated GFP-ORF6 was generated (Fig. 7C). ORF6 was predicted to carry a mono-partite NLS located between aa 1,116 and 1,124 near the C-terminal region (Fig. 7C). Mutations at the basic residues (KKRK to AARK) of the NLS motif in GFP-ORF6 completely abolished its nuclear translocation (Fig. 7C, NLS-mt). In coexpression experiments, we found that GFP-ORF6(NLS-mt) colocalized only with F-ORF40/41 but not F-ORF59 (Fig. 7D; *r* = 0.56 ± 0.13 and *r* = –0.25 ± 0.12, respectively). These results suggested that ORF40/41 could be the only core replication protein that interacts with ORF6 (Fig. 7A).

### Analysis of the protein-protein interactions among the viral core replication proteins by coimmunoprecipitation

**Fig 8:**
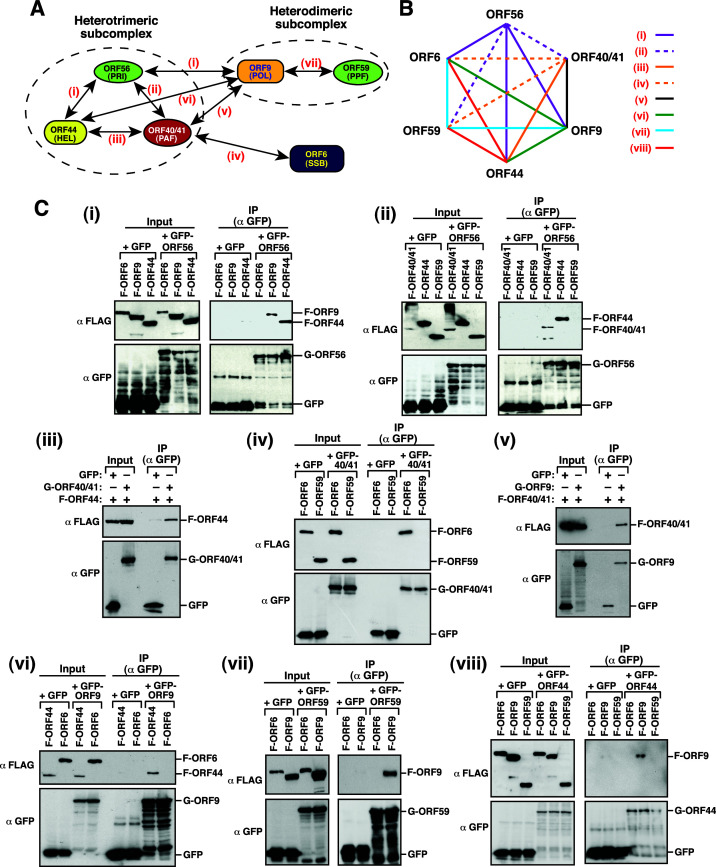
Analysis of the pairwise protein-protein interactions among the viral core replication proteins by coimmunoprecipitation. (**A**) A proposed protein-protein interaction network of the KSHV core replication complex. There are three major modules in this interaction network, including a heterotrimeric subcomplex, a heterodimric subcomplex, and the ORF6 subunit. Labels (i–vii) indicate the pairwise protein-protein interactions determined in coimmunoprecipitation (Co-IP) experiments shown in (**C**). (**B**) Schematic illustration of Co-IP experiments (i–viii) conducted in (**C**) using specific sets of viral core replication proteins. (**C**) Co-IP analysis of the indicated protein-protein interactions. Typically, 293T cells were cotransfected with the indicated expression plasmids for 24 h, and then, the protein lysates from these transfected cells were immunoprecipitated using anti-GFP antibody. After immunoprecipitation, the precipitated proteins were analyzed by Western blotting using anti-GFP and anti-FLAG antibodies. All Co-IP experiments were repeated at least twice with similar results.

Based on our confocal microscopic analysis, a protein-protein interaction network of the six KSHV-encoded core replication proteins was proposed (Fig. 8A). To further confirm the interactions between these core replication proteins, coimmunoprecipitation (Co-IP) experiments were performed using different combinations of the viral core replication proteins (summarized in Fig. 8B: i–viii). By using GFP-ORF56 as a target protein, we demonstrated that the core replication proteins including F-ORF9, F-ORF44, and F-ORF40/41 could be coimmunoprecipitated with GFP-ORF56 (Fig. 8C-i and -ii). However, both F-ORF6 and F-ORF59 could not be coimmunoprecipitated with GFP-ORF56 (Fig. 8C-i and -ii). For studying the interaction of ORF40/41 with the other core replication proteins, Co-IP experiments were performed using coexpression of GFP-ORF40/41 (or F-ORF40/41) with the other core replication proteins in 293T cells (Fig. 8C-iii, -iv, and -v). Besides the interaction between ORF40/41 and ORF56 (Fig. 8C-ii), our results demonstrated that ORF40/41 could specifically interact with ORF44, ORF6, and ORF9 but not ORF59 (Fig. 8C-iii, -iv, and -v). These results suggested that ORF40/41 could interact with all core replication proteins except for ORF59 (Fig. 8A).

As mentioned above, ORF9 was able to interact with ORF56 and ORF40/41 in Co-IP assays (Fig. 8B-i and B-v). We next examined the interaction of ORF9 with the other three core replication proteins including ORF44, ORF6, and ORF59 by Co-IP assays. Our data showed that F-ORF44, but not F-ORF6, could be coimmunoprecipitated with GFP-ORF9 (Fig. 8B-vi). Furthermore, Co-IP analysis also demonstrated the interaction between F-ORF9 and GFP-ORF59 (Fig. 8B-vii). Collectively, these results concluded that ORF9 could interact with all core replication proteins except for ORF6 (Fig. 8A).

Based on the results described above, ORF44 was shown to interact with ORF56, ORF40/41, and ORF9 (Fig. 8C-i, -iii, and -vi). However, we failed to detect the specific interaction between GFP-ORF44 and F-ORF6 or F-ORF59 by Co-IP assays in 293T cells (Fig. 8C-viii). These results supported that ORF44 could interact with ORF56, ORF40/41, and ORF9 but not ORF6 and ORF59 (Fig. 8A).

In the case of ORF59, our Co-IP assays demonstrated that ORF59 only interacted with ORF9 (Fig. 8C-vii) but not with ORF56, ORF40/41, ORF6, and ORF44 (Fig. 8C-ii, -iv, -vii, and -viii). Similarly, when the interaction between ORF6 and other core replication proteins was determined by Co-IP assays, we found that ORF6 only interacted with ORF40/41 (Fig. 8C-iv) but not with ORF56, ORF9, ORF59, and ORF44 (Fig. 8Ci, -vi, -vii, and -viii). Overall, our Co-IP results were consistent with data obtained from confocal fluorescence microscopy (Fig. 8A).

### Evaluating the assembly of the subunits of the helicase-primase subcomplex by the GAL4/VP16-based reporter system

**Fig 9:**
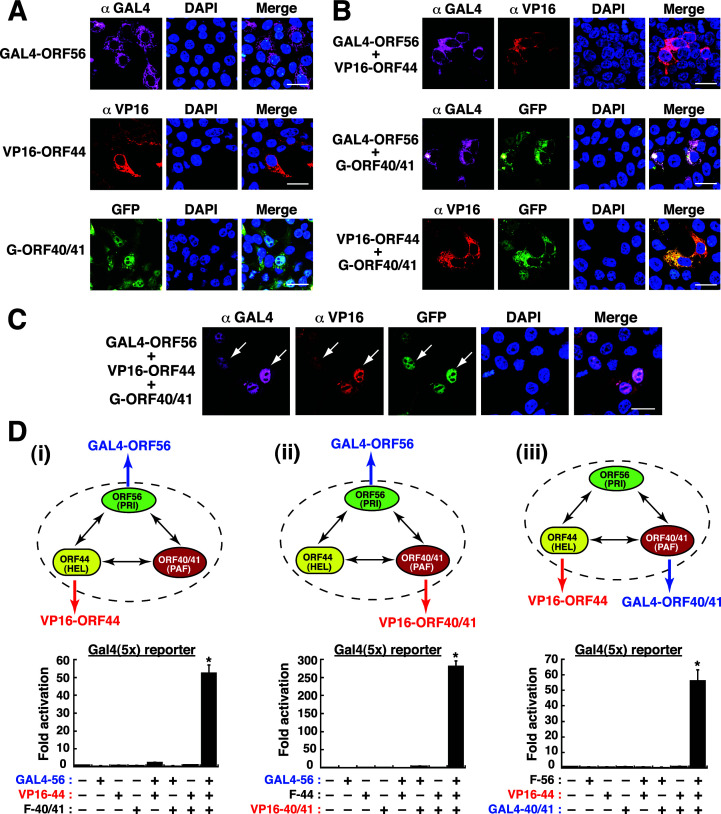
Assessment of the formation of the ORF56–ORF44–ORF40/41 subcomplex using a two-hybrid (GAL4/VP16) reporter assay. (**A**) Confocal microscopic analysis of the subcellular localization of GAL4-ORF56, VP16-ORF44, and GFP-ORF40/41 individually expressed in 293T cells. Scale bars, 20 µM. (**B**) Confocal microscopic analysis of the pairwise interactions among GAL4-ORF56, VP16-ORF44, and GFP-ORF40/41 in 293T cells. Scale bars, 20 µM. (**C**) Confocal microscopic analysis of the colocalization of GAL4-ORF56, VP16-ORF44, and GFP-ORF40/41 in the nucleus of 293T cells. Scale bars, 20 µM. All of the experiments in (A–C) were independently repeated at least three times with similar results. (**D**) Transcriptional activation of a Gal4(5×)-driven reporter construct by (i) GAL4-ORF56, VP16-ORF44, and F-ORF40/41; (ii) GAL4-ORF56, F-ORF44, and VP16-ORF40/41; or (iii) F-ORF56, VP16-ORF44, and GAL4-ORF40/41 in 293T cells. Data are presented as means ± SD (*n* = 3). *P < 0.01, for results compared to those from the empty vector group.

Due to some limitations in Co-IP assay (e.g., largely depending on the strength of the underlying protein-protein interaction) and in confocal microscopy (e.g., limited resolution for the detection of specific protein-protein interactions), we attempted to use mammalian two-hybrid (GAL4/VP16)-based reporter system to characterize the assembly of the multi-subunit protein subcomplex. In this assay system, the DNA-binding domain of a yeast transcription factor GAL4 and the activation domain of HSV-1 VP16 were separately fused to different core replication proteins. A physical contact between the resultant GAL4- and VP16-fused proteins in the nucleus might lead to the reconstitution of a functional transactivator able to activate the reporter construct containing five GAL4-binding sites [pGal4(5×)-Luc]. For studying the assembly of the heterotrimeric helicase-primase subcomplex, the GAL4 DNA-binding domain was first fused to the N-terminus of ORF56 (designated as GAL4-ORF56), and the VP16 activation domain was fused to the N-terminus of ORF44 (designated as VP16-ORF44) (Fig. 9; Fig. S3). As expected, confocal microscopic analysis revealed that the GAL4-ORF56 and VP16-ORF44 fusion constructs, when expressed individually or co-expressed together, were localized in the cytoplasm (Fig. 9A and B). Only coexpression of GAL4-ORF56, VP16-ORF44, and GFP-ORF40/41 together in 293T cells allowed the nuclear transport of all these proteins (Fig. 9C). When various combinations of the plasmids expressing GAL4-ORF56, VP16-ORF44, and F-ORF40/41 were cotransfected with pGal4(5×)-Luc in 293T cells, we found that only cells transfected with all three expression plasmids showed robust reporter gene activation up to 55-fold (Fig. 9D-i). These results reflected the assembly of the trimeric subcomplex containing GAL4-ORF56, VP16-ORF44, and F-ORF40/41 in the nucleus of transfected cells.

To verify the consistency of this assay system, another fusion construct VP16-ORF40/41 was combined with GAL4-ORF56 in the experiments (Fig. 9D-ii). As shown in Fig. 9D-ii, different sets of the plasmids encoding GALF4-ORF56, VP16-ORF40/41, and F-ORF44 were cotransfected with the reporter plasmid into 293T cells. Our results showed that only coexpression of GAL4-ORF56, VP16-ORF40/41, and F-ORF44 together in cells led to an abundant enhancement of luciferase activity (up to 280-fold). Similarly, the fusion constructs including GAL4-ORF40/41 and VP16-ORF44 were also included in the assay system (Fig. 9D-iii). Consistently, only cells expressing GAL4-ORF40/41, VP16-ORF44, and F-ORF56 were able to abundantly activate the reporter expression (Fig. 9D-iii, up to 60-fold). Collectively, these results strongly suggested that the GAL4/VP16-based reporter system would be a useful tool to evaluate the formation of the multi-subunit subcomplex.

### Determining the interactions among ORF9, ORF59, and ORF6 by the GAL4/VP16-based reporter system

**Fig 10:**
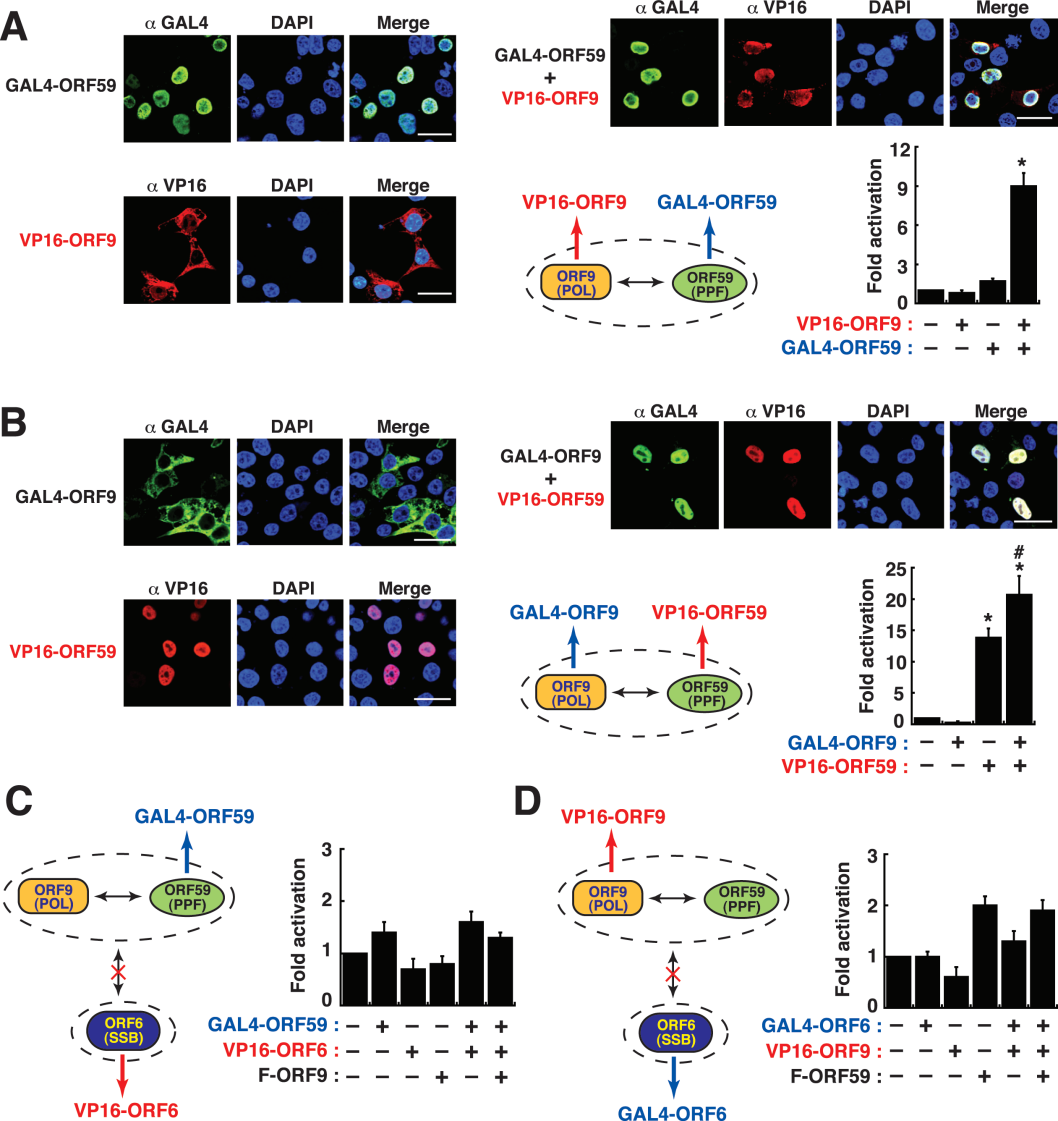
Analysis of the formation of the ORF9–ORF59 subcomplex and the possible interaction between the ORF9–ORF59 subcomplex and ORF6 using a two-hybrid (GAL4/VP16) reporter assay. (**A**) Confocal microscopic analysis and GAL4/VP16-mediated reporter analysis of the interaction between GAL4-ORF59 and VP16-ORF9. Scale bars in the confocal microscopic images represent 20 µM. The confocal microscopy experiments were performed at least three times independently with similar results. The results from reporter assays are expressed as means ± SD (*n* = 3). *P < 0.01, for results compared to those from the empty vector group. (**B**) Confocal microscopic analysis and GAL4/VP16-mediated reporter analysis of the interaction between GAL4-ORF9 and VP16-ORF59. Scale bars in the confocal microscopic images represent 20 µM. The confocal microscopy experiments were performed at least three times independently with similar results. The results from reporter assays are expressed as means ± SD (*n* = 3). *P < 0.01, for results compared to those from the empty vector group; #, *P* < 0.01, for results compared to those from the VP16-ORF59-transfected group. (**C**) Effect of the coexpression of GAL4-ORF59, VP16-ORF6, and/or F-ORF9 on transcriptional activation of a Gal4(5×)-driven reporter construct. Data are presented as means ± SD (*n* = 3). (**D**) Effect of the coexpression of GAL4-ORF6, VP16-ORF9, and/or F-ORF59 on transcriptional activation of a Gal4(5×)-driven reporter construct. Data are presented as means ± SD (*n* = 3).

To apply the GAL4/VP16-based reporter system to analyze the interaction between ORF59 and ORF9, the fusion constructs GAL4-ORF59 and VP16-ORF9 were generated (Fig. S3). Confocal microscopic analysis showed that VP16-ORF9 alone was localized in the cytoplasm, whereas coexpression of GAL4-ORF59 allowed the nuclear transport of VP16-ORF9 (Fig. 10A). For the reporter assay, activation of the pGal4(5×)-Luc reporter gene was detected only in the presence of both VP16-ORF9 and GAL4-ORF59 in cells (Fig. 10A). In a similar way, the plasmids encoding GAL4-ORF9 and VP16-ORF59 were also included in the reporter assay (Fig. 10B). However, we noticed that cells expressing VP16-ORF59 alone showed a high background level of reporter expression (Fig. 10B). The high background reporter activation might be due to a high intrinsic DNA-binding activity of ORF59 (25, 27). Thus, nonspecific DNA binding of VP16-ORF59 to the reporter plasmid DNA probably caused the high-level reporter activation. Despite having a high-level reporter activation mediated by VP16-ORF59, coexpression of GAL4-ORF9 with VP16-ORF59 could further increase the reporter activation of pGal4(5×)-Luc (Fig. 10B).

According to the results from confocal microscopic experiments and Co-IP experiments, ORF9 or ORF59 alone could not interact with ORF6 (Fig. 8). To investigate whether the ORF9–ORF59 subcomplex could interact with ORF6 in the nucleus, the ORF6 fusion constructs including VP16-ORF6 and GAL4-ORF6 were individually used in the reporter assay (Fig. 10C and D). Our results showed that coexpression of VP16-ORF6 with GAL4-ORF59 and F-ORF9 (Fig. 10C) or coexpression of GAL4-ORF6 with VP16-ORF9 and F-ORF59 in 293T cells (Fig. 10D) could not activate the reporter gene expression. These findings implicated that there was no direct interaction between ORF6 and the ORF9–ORF59 subcomplex in the nucleus.

### Investigating the association between the helicase-primase subcomplex and the ORF6 subunit by the GAL4/VP16-based reporter assay

**Fig 11:**
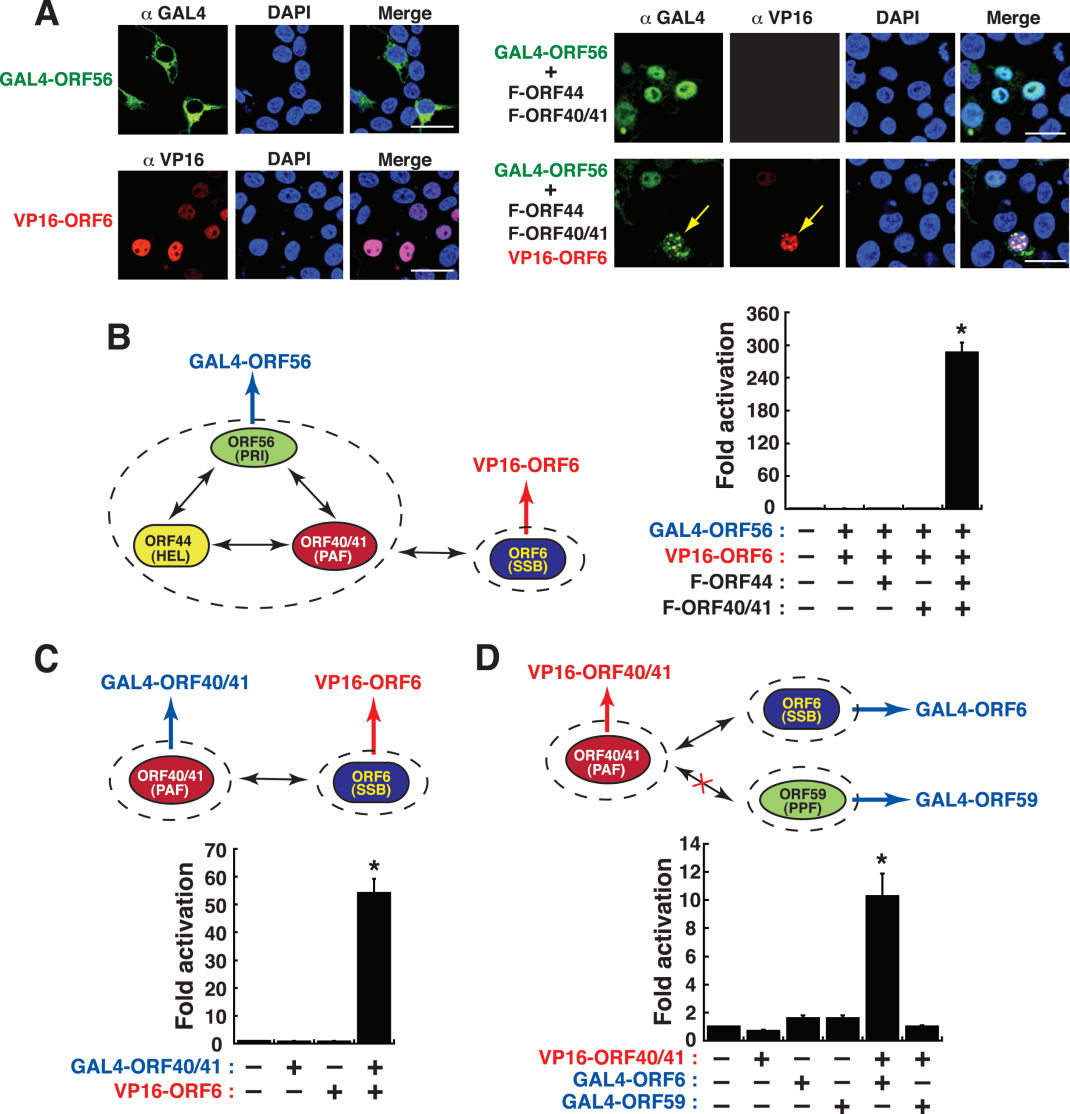
Evaluation of the association between the ORF56–ORF44–ORF40/41 subcomplex and ORF6 using a two-hybrid (GAL4/VP16) reporter assay. (**A**) Confocal microscopic analysis of the assembly of the tetrameric subcomplex containing GAL4-ORF56, F-ORF44, F-ORF40/41, and VP16-ORF6. As noted, when 293T cells were coexpressing F-ORF44, F-ORF40/41, and VP16-ORF6 (yellow arrows), GAL4-ORF56 formed puncta in the cell nucleus. Scale bars, 20 µM. The experiments were repeated three times independently with similar results. (**B**) Transcriptional activation of a Gal4(5×)-driven reporter construct by coexpression of GAL4-ORF56, F-ORF44, F-ORF40/41, and VP16-ORF6. Data are presented as means ± SD (*n* = 3). *P < 0.01, for results compared to those from the empty vector group. (**C**) Activation of a Gal4(5×)-driven reporter construct by coexpression of GAL4-ORF40/41 and VP16-ORF6. Data are presented as means ± SD (*n* = 3). *P < 0.01, for results compared to those from the empty vector group. (**D**) Activation of a Gal4(5×)-driven reporter construct by coexpression of VP16-ORF40/41 and GAL4-ORF6 but not by coexpression of VP16-ORF40/41 and GAL4-ORF59. Data are presented as means ± SD (*n* = 3). *P < 0.01, for results compared to those from the empty vector group.

As proposed in Fig. 8A, the trimeric ORF56–ORF44–ORF40/41 subcomplex could interact with ORF6 via the ORF40/41 subunit. To verify the association of these protein subunits, the fusion constructs including GAL4-ORF56 and VP16-ORF6, along with F-ORF44 and F-ORF40/41, were initially coexpressed and tested in 293T cells (Fig. 11A). Confocal microscopic analysis revealed that coexpression of F-ORF44, F-ORF40/41, and VP16-ORF6 allowed the formation of GAL4-ORF56 puncta in the nucleus (Fig. 11A). When different combinations of the plasmids encoding GAL4-ORF56, VP16-ORF6, F-ORF44, and F-ORF40/41 were cotransfected with pGal4(5×)-Luc into 293T cells (Fig. 11B), we found that only cotransfection of all four expression plasmids in cells resulted in an abundant reporter activation (Fig. 11B, up to 280-fold). Since ORF40/41 alone could interact with ORF6 as shown in confocal microscopic experiments and in co-IP assays (Fig. 7; Fig. 8B-iv), we examined whether coexpression of GAL4-ORF40/41 and VP16-ORF6 conferred reporter activation. Our results clearly demonstrated that coexpression of GAL4-ORF40/41 and VP16-ORF6 together sufficiently increased the reporter activity by 56-fold (Fig. 11C). On the other hand, we also tested the effect of the combination of VP16-ORF40/41 and GAL4-ORF6 (or GAL4-ORF59) on the reporter activation (Fig. 11D). Our results showed that coexpression of VP16-ORF40/41 and GAL4-ORF6, but not coexpression of VP16-ORF40/41 and GAL4-ORF59, led to activation of the reporter expression (Fig. 11D). Taken together, our results suggested that (i) the viral core replication proteins including ORF56, ORF44, ORF40/41, and ORF6 could form a stable tetrameric subcomplex in the nucleus; (ii) ORF40/41 within the helicase-primase subcomplex was the key subunit that interacts with ORF6; (iii) both ORF40/41 and ORF6 potentially formed another stable subcomplex in the nucleus; (iv) ORF40/41 could not interact with ORF59 in the nucleus.

### Analyzing the association between the helicase-primase subcompex and the replisome subcomplex

**Fig 12:**
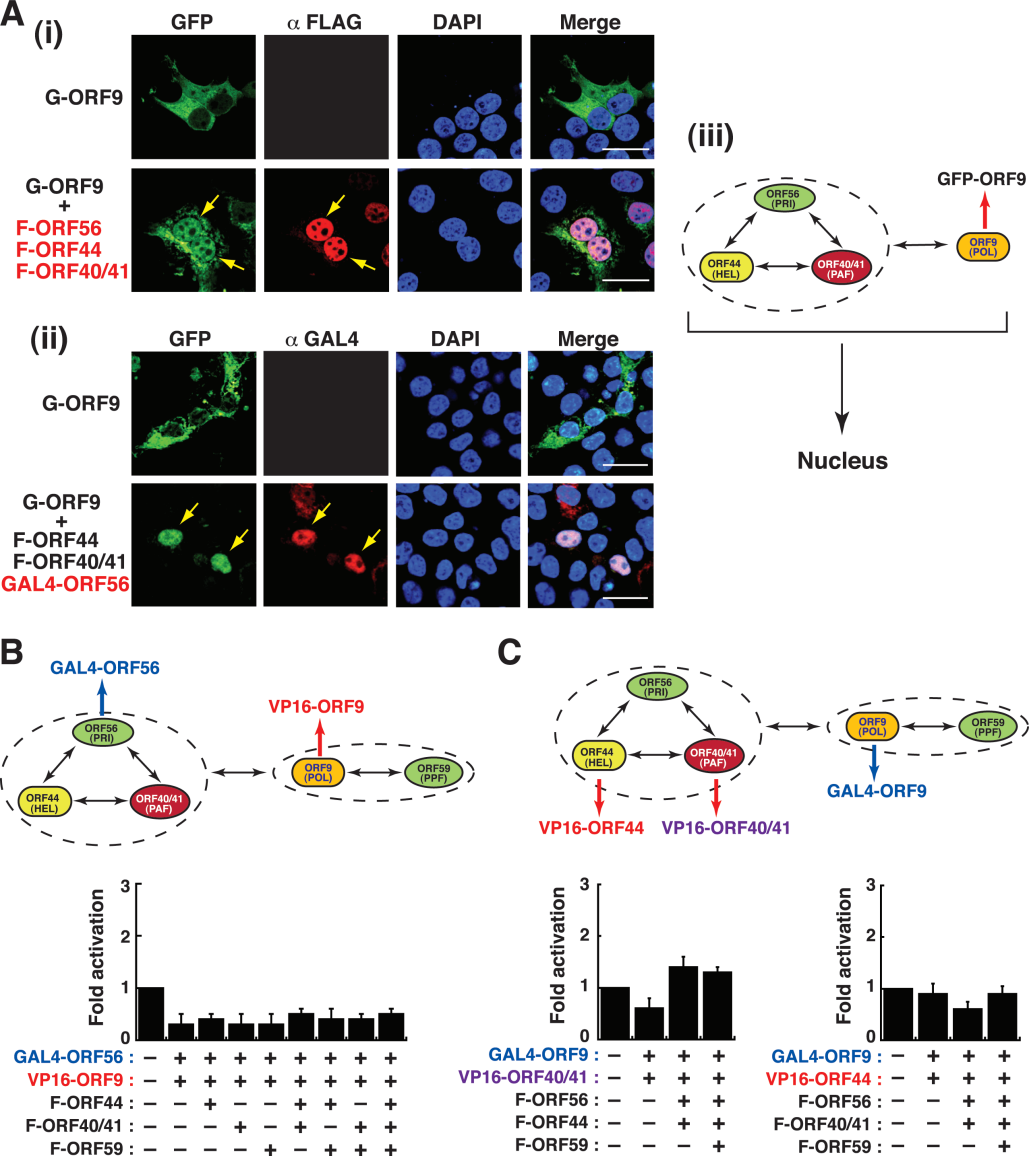
Evaluation of the association between the ORF56–ORF44–ORF40/41 subcomplex and ORF9 (or the replisome subcomplex) using confocal microscopy and a two-hybrid (GAL4/VP16) reporter assay. (**A**) Confocal microscopic analysis of the association between the ORF56–ORF44–ORF40/41 subcomplex and GFP-ORF9 in 293T cells. Yellow arrows in (i) and (ii) indicate that GFP-ORF9 was conveyed into the nucleus in the presence of F-ORF44, F-ORF40/41, and F-ORF56 (or GAL4-ORF56). Scale bars, 20 µM. All the experiments were repeated at least three times with similar results. The panel (iii) shows the proposed model for the association between the ORF56–ORF44–ORF40/41 subcomplex and ORF9. (**B**) Analysis of the association between the helicase-primase subcomplex and the replisome subcomplex by using the combination of GAL4-ORF56 and VP16-ORF9 in the two-hybrid (GAL4/VP16) reporter system. Data are expressed as means ± SD (*n* = 3). (**C**) Analysis of the association between the helicase-primase subcomplex and the replicase subcomplex by using the combination of VP16-ORF44 and GAL4-ORF9 or the combination of VP16-ORF40/41 and GAL4-ORF9 in the two-hybrid (GAL4/VP16) reporter system. Data are expressed as means ± SD (*n* = 3).

As described above, all the subunits of the helicase-primase subcomplex were able to individually interact with ORF9 (Fig. 5 and 8). Due to the fact that the helicase-primase subcomplex could enter the nucleus, we sought to determine whether the helicase-primase subcomplex could convey ORF9 to the nucleus. To test this possibility, we first examined the subcellular localization of GFP-ORF9 in the presence of F-ORF56, F-ORF44, and F-ORF40/41 (Fig. 12A-i). Confocal microscopy analysis showed that GFP-ORF9 could be partially transported into the nucleus in the presence of F-ORF56, F-ORF44, and F-ORF40/41 (Fig. 12A-i). Similarly, when GFP-ORF9 was coexpressed with F-ORF44, F-ORF40/41, and GAL4-ORF56, both GFP-ORF9 and GAL4-ORF56 were colocalized in the nucleus (Fig. 12A-ii). These results, therefore, implied that ORF9 could be transported into the nucleus via the helicase-primase subcomplex (Fig. 12A-iii).

To further investigate the association between the ORF56–ORF44–ORF40/41 subcomplex and the ORF9–ORF59 subcomplex, we used “GAL4-ORF56” as a representative of the ORF56–ORF44–ORF40/41 subcomplex and “VP16-ORF9” as a representative of the ORF9–ORF59 subcomplex in the GAL4/VP16-mediated reporter assay (Fig. 12B). Unfortunately, we failed to detect significant reporter activation when both GAL4-ORF56 and VP16-ORF9 were coexpressed with other core replication proteins (Fig. 12B). Besides using the combination of GAL4-ORF56 plus VP16-ORF9, different combinations including VP16-ORF44 plus GAL4-ORF9 or VP16-ORF40/41 plus GAL4-ORF9 were also utilized in the reporter system (Fig. 12C). However, we still could not detect a significant reporter activation using these fusion protein combinations (Fig. 12C). It could be possible that the assembly of the multi-subunit protein complex might cause steric hindrance, thereby affecting the transactivation function of both VP16- and GAL4-fusion constructs.

### Effect of radicicol on the formation of both the helicase-primase and replicase subcomplexes

**Fig 13:**
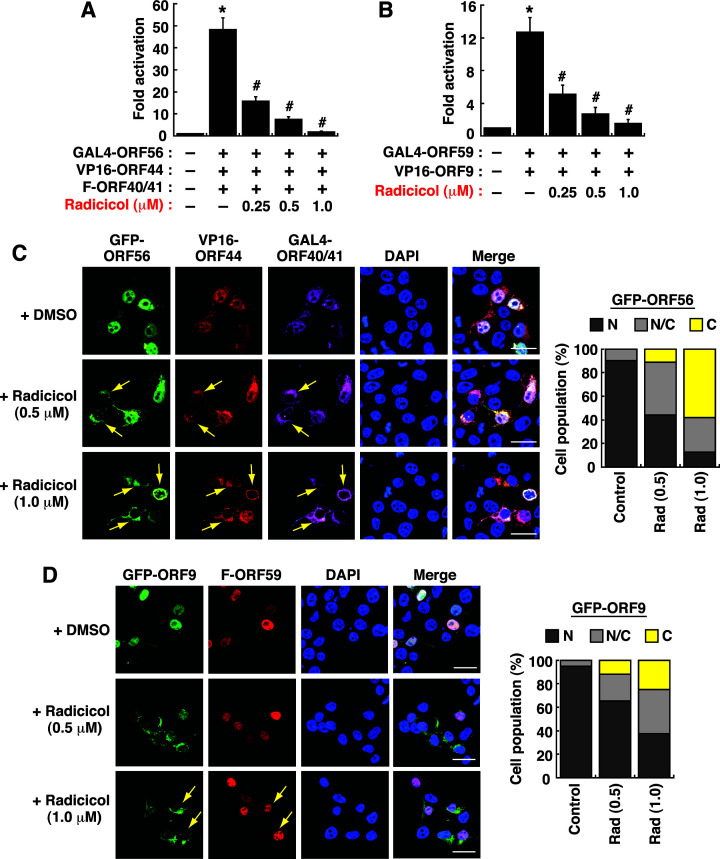
Inhibition of the formation of the helicase-primase and replisome subcomplexes by radicicol. (**A**) Effects of increasing amounts of radicicol on the reporter activation mediated by coexpression of GAL4-ORF56, VP16-ORF44, and F-ORF40/41 in 293T cells. Data are presented as means ± SD (*n* = 3). *P < 0.01, for results compared to those from the empty vector group; #*P* < 0.01, for results compared to those from the untreated ORF56–ORF44–ORF40/41 group. (**B**) Effects of increasing amounts of redicicol on the reporter activation mediated by GAL4-ORF59 and VP16-ORF9 in 293T cells. Data are presented as means ± SD (*n* = 3). *P < 0.01, for results compared to those from the empty vector group; #*P* < 0.01, for results compared to those from the untreated ORF59–ORF9 group. (**C**) Effects of radicicol on the subcellular localization of components of the helicase-primase subcomplex in 293T cells. In the experiments, 293T cells cotransfected with the plasmids encoding GFP-ORF56, VP16-ORF44, and GAL4-ORF40/41 were treated with DMSO (used as a solvent) or radicicol (0.5 and 1.0 µM) for 24 h, and then, the subcellular localization of each protein in transfected cells was analyzed by confocal microscopy. Scale bars, 20 µM. The subcellular localization patterns of GFP-ORF56 (acting as a representative) in different treatment groups were calculated and plotted in the right panel (*n* = 3). N, nucleus; N/C, both nucleus and cytoplasm; C, cytoplasm. (**D**) Effects of radicicol on the subcellular localization of components of the replisome subcomplex in 293T cells. In the experiments, 293T cells cotransfected with the plasmids encoding GFP-ORF9 and F-ORF59 were treated with DMSO (used as a solvent) or radicicol (0.5 and 1.0 µM) for 24 h, and the subcellular localization of each protein in transfected cells was analyzed by confocal microscopy. Scale bars, 20 µM. Changes in the subcellular localization of GFP-ORF9 in different treatment groups were shown in the right panel (*n* = 3). N, nucleus; N/C, both nucleus and cytoplasm; C, cytoplasm. As noted, radicicol treatment did not affect the nuclear transport of F-ORF59.

To study whether small molecules could specifically disrupt the assembly or nuclear transport of the viral helicase-primase subcomplex and the replisome subcomplex, a compound library was screened by using the GAL4/VP16-based reporter system. We here found that radicicol, an inhibitor of heat shock protein 90 (Hsp90), could significantly inhibit the reporter activation induced by coexpression of GAL4-ORF56, VP16-ORF44, and F-ORF40/41 (Fig. 13A) and by coexpression of GAL4-ORF59 and VP16-ORF9 (Fig. 13B) in a dose-dependent manner. It should be noted that radiciol at the concentrations used in the study (0.25–1 µM) displayed only weak cytotoxic activity on 293T cells (Fig. S4). To further verify the effect of radicicol on the assembly and nuclear transport of the ORF56–ORF44–ORF40/41 subcomplex and the ORF59–ORF9 subcomplex, the subcellular localization of individual core replication proteins in 293T cells was examined by confocal fluorescence microscopy. When GFP-ORF56, VP16-ORF44, and GAL4-ORF40/41 were coexpressed in 293T cells, we found that treatment with increasing amounts of radicicol (0.5 and 1 µM) greatly increased the cytoplasmic retention of these core replication proteins in cells (Fig. 13C). On the other hand, when GFP-ORF9 and F-ORF59 were coexpressed in 293T cells, treatment with radicicol specifically affected the nuclear localization of GFP-ORF9 but not F-ORF59 (Fig. 13D). These results implied that Hsp90 was required for the protein-protein interaction and/or the nuclear transport of both the helicase-primase and replisome subcomplexes.

## DISCUSSION

Although the lytic DNA replication of herpesviruses has been studied for several decades, the protein-protein interactions of herpesviral DNA replication proteins are not yet fully understood. In this report, we characterize the protein-protein interactions of six KSHV-encoded core replication proteins by using different methods, and a complete protein-protein interaction network of these conserved core replication proteins is proposed. This study may be important not only to deepen our knowledge about the assembly of the viral DNA replication complex but also to provide opportunities to develop new strategies against viral propagation.

### Coordinate regulation of the assembly of KSHV core replication proteins

During the KSHV lytic cycle, the expression and the interaction of the viral core replication components must be tightly controlled to allow the correct assembly of a functional replication complex in the cell nucleus. Although the lytic DNA replication of KSHV takes place within the cell nucleus, we here showed that three viral replication components with enzymatic activity including ORF56, ORF44, and ORF9 when expressed individually, were restricted to the cytoplasm (Fig. 1). It is possible that the cytoplasmic retention of specific viral replication components may be a key control mechanism to avoid unlicensed viral DNA replication when other necessary core replication components are not yet available during the viral lytic cycle or when leaky expression of these viral proteins occurs upon some stress conditions. In our research, we demonstrated that the nuclear translocation of OR56 and ORF44 requires ORF40/41 (forming a trimeric subcomplex). Additionally, consistent with previous studies (26), we confirmed that the nuclear translocation of ORF9 requires ORF59 (forming a dimeric subcomplex).

Like HSV-1 and EBV, the KSHV core replication complex could be generally divided into three modules including the ORF56–ORF44–ORF40/41 trimeric subcomplex, the ORF9–ORF59 dimeric subcomplex, and the ORF6 subunit (Fig. 8A). To monitor the intermediates in the assembly of the multi-subunit replication complex, we chose ORF56 as a target to analyze its subcellular localization along with other core replication proteins (Fig. 2). According to our results, ORF56 initially entered the nucleus in the presence of ORF44 and ORF40/41, leading to the formation of the helicase-primase subcomplex. The resultant ORF56–ORF44–ORF40/41 subcomplex in the nucleus may interact with the ORF6 subunit and/or the ORF9–ORF59 heterodimeric subcomplex. Interestingly, coexpression of the ORF56–ORF44–ORF40/41 subcomplex with ORF6 could cause the formation of ORF56 puncta in the nucleus. Particularly, these ORF56 nuclear puncta observed in transfected cells significantly colocalized with the signals of γH2AX, 53BP1, or RPA foci (Fig. 3). Since ORF6 in the nucleus may preferentially bind to single-stranded DNA intermediates derived from cellular DNA replication, repair, or recombination, we speculate that the recruitment of the helicase-primase subcomplex to these DNA sites through ORF6 may trigger DNA replication stress, ultimately leading to double-stranded DNA breaks and significant DNA damage. In agreement with previous observations, several studies have shown that lytic induction of KSHV could lead to activation of the DNA damage response (DDR) pathway including increased phosphorylation of H2AX (36 – 38). In future studies, the detailed relationship among the viral core replication subcomplex, cellular DNA repair enzymes, and the potential DNA damage sites needs to be further characterized. In contrast, we did not find the punctate pattern of ORF56 when the ORF56–ORF44–ORF40/41 subcomplex was coexpressed with the ORF9–ORF59 subcomplex. As compared to the coexpression of four core replication proteins (including ORF56, ORF44, ORF40/41, and ORF6), the coexpression of all six core replication proteins significantly increased the number of ORF56 puncta in the nucleus (Fig. 2B).

### Protein-protein interaction networks of KSHV-encoded core replication components

Detailed characterization of the pairwise protein-protein interactions revealed that all three components within the helicase-primase subcomplex could interact with each other (Fig. 8). Furthermore, we propose that the association of the helicase-primase subcomplex with ORF6 in the nucleus is mediated through the interaction between ORF40/41 and ORF6 (Fig. 8A and 11). For the association between the helicase-primase subcomplex and the replisome subcomplex, our results showed that each component within the helicase-primase subcomplex could individually interact with ORF9 but not with ORF59 (Fig. 8A). It is possible that during the viral lytic DNA synthesis, ORF9 may dynamically interact with the components of the helicase-primase subcomplex to coordinate DNA synthesis of the leading and lagging strands. On the other hand, we did not detect any significant interaction between the replisome subcomplex and ORF6. Besides the well-conserved helicase-primase and replisome subcomplexes in the nucleus, we additionally found that several distinct, stable subcomplexes exist in the nucleus. These subcomplexes include (i) the dimeric subcomplex containing ORF40/41 and ORF6 (Fig. 11); (ii) the tetromeric subcomplex containing ORF56, ORF44, ORF40/41, and ORF6 (Fig. 11); and (iii) the tetromeric subcomplex containing ORF56, ORF44, ORF40/41, and ORF9 (Fig. 12). Noteworthily, the formation of the latter subcomplex ORF56–ORF44–ORF40/41–ORF9 could be of potential importance because the helicase-primase subcomplex may have the ability to convey ORF9 to the nucleus when ORF59 is absent (Fig. 12).

Although a protein-protein interaction network of six KSHV-encoded core replication proteins is proposed here, the interaction network still needs to be further confirmed in the context of KSHV infection. To achieve the goal, both the preparation of reliable antibodies specific to endogenous core replication proteins and the creation of a set of PEL cell lines with specific gene knockout in KSHV genome may be helpful for experimental investigations. In addition to the six KSHV replication core proteins, the assembly of a complete functional replication complex is believed to require other viral proteins (such as ORF50 and K8) or cellular proteins as well as the *cis*-acting lytic origin (oriLyt). The interrelationship of these replication components remains to be further determined. Moreover, since dimerization or multimerization of specific core replication proteins may be necessary for the assembly of the replication complex, future experiments may need to determine whether certain replication proteins are able to form dimers or multimers.

In the study, different methods including confocal fluorescence microscopy, Co-IP, and the GAL4/VP16-mediated reporter system were used to determine the protein-protein interactions of KSHV core replication proteins. However, these methods individually have certain limitations. For instance, low-resolution images taken by confocal fluorescence microscopy might lead to false data interpretation. Although Co-IP assay is considered a gold standard method for studying the protein-protein interaction, the low-affinity protein-protein interactions might not be easily detected. Herein, we attempted to establish the GAL4/VP16-based reporter system for probing interactions among subunits of different subcomplexes. This approach is a fast and highly sensitive assay, which may serve as a screening tool for identifying small compounds that inhibit the protein-protein interactions of the viral core replication proteins. Although the GAL4/VP16-mediated reporter system is useful to determine physiologically relevant protein-protein interactions, there are two major limitations for this approach. First, this established method is not suitable for detecting the assembly intermediates that are localized in the cytoplasm (such as heterodimeric subcomplexes: ORF56–ORF44, ORF56–ORF9, and ORF44–ORF9). Second, due to the fact that the formation of multi-subunit subcomplexes might cause steric hindrance of GAL4- and/or VP-16-containing proteins, this method might have a problem to detect the assembly of certain subcomplexes (e.g., the ORF56–ORF44–ORF40/41–ORF9 subcomplex) and the full replication complex (Fig. 12).

### Role of Hsp90 in the formation of both the helicase-primase and replicase subcomplexes of KSHV

Hsp90 is an evolutionally conserved molecular chaperone (39, 40). Previous studies have shown that Hsp90 plays a critical role in the nuclear transport of the HSV-1 DNA polymerase UL30 and the EBV DNA polymerase BALF5, two homologs of the KSHV ORF9 (41, 42). In the case of the HSV-1 UL30, it is normally localized in the nucleus when expressed alone (43); however, treatment of the Hsp90 inhibitor geldanamycin blocked its nuclear transport (41). Unlike the HSV-1 UL30, the EBV DNA polymerase BALF5 is localized in the cytoplasm when expressed alone. The nuclear transport of the EBV BALF5 requires the polymerase processivity factor BMRF1 (42). Pharmacological inhibition or genetic knockdown of Hsp90 markedly prevented the nuclear transport of the EBV BALF5 even in the presence of BMRF1 (42). It should be noted that inhibition of the Hsp90 activity did not affect the nuclear transport of BMRF1 (42). By using the GAL4/VP16-based reporter assay, we found that the Hsp90 inhibitor radicicol was able to reduce the reporter activation mediated by the combination of GAL4-ORF59 and VP16-ORF9 in a dose-dependent manner (Fig. 13B). Confocal microscopic analysis further demonstrated that radicicol significantly inhibited the nuclear transport of ORF9 but not the nuclear transport of ORF59. These results were similar to those obtained from the study of the EBV BALF5 and BMRF1 (42). Our findings, therefore, conclude that Hsp90 is involved in the interaction between ORF9 and ORF59. On the other hand, our studies also revealed that radicicol treatment could markedly inhibit the reporter activation induced by the combination of GAL4-ORF56, VP16-ORF44, and F-ORF40/41 in the GAL4/VP16-based reporter assay (Fig. 13A). Confocal microscopic analysis demonstrated that radicicol could block the nuclear translocation of all the helicase-primase subcomplex components. These results suggest that Hsp90 may play critical roles in the protein-protein interactions of the helicase-primase subcomplex and/or the subsequent nuclear transport of this subcomplex. Although the detailed mechanisms underlying the role of Hsp90 in the assembly of both the KSHV helicase-primase and replisome subcomplexes still remain unclear, we think that Hsp90 could be a common factor essential for the assembly of the core replication complex in all of herpesviruses. In addition to Hsp90 inhibitors, our experiments have also identified several bioactive small molecules that could inhibit the reporter activation mediated by coexpression of GAL4-ORF56, VP16-ORF44, and F-ORF40/41 or by coexpression of GAL4-ORF59 and VP16-ORF9. Further research is needed to elucidate the mechanism of action of these identified bioactive compounds in affecting the assembly of the KSHV core replication complex.

In summary, we characterize the protein-protein interactions of six KSHV-encoded core replication proteins by using different methods and a complete protein-protein interaction network of these viral core replication proteins is proposed. In addition to the well-conserved helicase-primase and replicase subcomplexes, several stable assembly intermediates are also identified in the cell nucleus. Furthermore, we show that Hsp90 plays significant roles in the assembly of the KSHV core replication complex.

## MATERIALS AND METHODS

### Cell cultures, transfections, and chemical reagents

The human embryonic kidney cell line 293T (44) was cultured in high-glucose Dulbecco’s modified Eagle’s medium (no. 11965084; Thermo Fisher Scientific) supplemented with 10% fetal bovine serum (FBS; no. 10437028; Thermo Fisher Scientific). The primary effusion lymphoma cell line HH-B2 was cultured in RPMI 1640 medium (no. 11875085; Thermo Fisher Scientific) supplemented with 15% FBS. Transfection experiments were performed using the Lipofectamine 2000 Transfection Reagent (no. 11668019; Thermo Fisher Scientific) according to the manufacturer’s instructions. To induce the KSHV lytic cycle in HH-B2 cells, cells were treated with 3 mM sodium butyrate (no. B5887; Sigma-Aldrich). In certain experiments, different concentrations of radicicol (no. R2146; Sigma-Aldrich), an inhibitor of Hsp90, were added to cultured cells.

### Plasmid construction

To generate the expression plasmids encoding ORF6, ORF9, ORF40/41, ORF44, ORF56, and ORF59, the corresponding DNA fragments amplified by PCR were cloned into pFLAG-CMV-2 (no. E7398; Sigma-Aldrich) or into pEGFP-C2 (no. 6083-1; Clontech). The resultant plasmids were designated pCMV-F-ORF6, pCMV-F-ORF9, pCMV-F-ORF40/41, pCMV-F-ORF44, pCMV-F-ORF56, and pCMV-F-ORF59, as well as pCMV-GFP-ORF6, pCMV-GFP-ORF9, pCMV-GFP-ORF40/41, pCMV-GFP-ORF44, pCMV-GFP-ORF56, and pCMV-GFP-ORF59. The NLS-mutated constructs of GFP-ORF6 and GFP-ORF59 were created in pCMV-GFP-ORF6 or pCMV-GFP-ORF59 using QuickChang site-directed mutagenesis kit (no. 200524; Agilent Technologies). The expression plasmids encoding GAL4-ORF56, GAL4-ORF40/41, GAL4-ORF6, GAL4-ORF9, and GAL4-ORF59 were generated by inserting the the coding region of the GAL4 DNA-binding domain into pCMV-F-ORF56, pCMV-F-ORF40/41, pCMV-F-ORF6, pCMV-F-ORF9, and pCMV-ORF59, respectively. The expression plasmids encoding VP16-ORF44, VP16-ORF40/41, VP16-ORF6, VP16-ORF9, VP16-ORF59 were generated by inserting the coding region of the VP16 activation domain into pCMV-F-ORF44, pCMV-F-ORF40/41, pCMV-F-ORF6, pCMV-F-ORF9, and pCMV-F-ORF59, respectively. To construct the reporter plasmid pGal4(5×)-Luc, the DNA fragment containing five GAL4 binding sites and an adenovirus E1b minimal promoter from pG5CAT (no. V006134; Clontech) were cloned into pGL3-Basic (no. E1751; Promega).

### Western blot analysis

Detailed procedures for Western blotting analysis were mentioned previously (45). Briefly, cells were lysed in either the Laemmli sample buffer containing 62.5 mM Tris-HCl (pH6.8), 2% sodium dodecyl sulfate (SDS), 10% glycerol, and 5% 2-mercaptoethanol, or the coimmunoprecipitation assay buffer containing 50 mM Tris-HCl (pH 7.6), 150 mM NaCl, 1 mM EDTA, 1% Triton X-100, and 1 mM phenylmethylsulfonyl fluoride. Protein lysates were resolved on 8%–10% SDS-polyacrylamide gel and then transferred onto polyvinylidene difluoride (PVDF) membranes (no. IEVH85R: Millipore). The membranes were incubated with antibodies specific to the FLAG tag (no. A8592; Sigma), GFP (no. G1544; Sigma), GAL4 (sc-510; Santa Cruz), VP16 (sc-7545; Santa Cruz), or actin (no. sc-47778; Santa Cruz). After extensive washing, the membranes were incubated with suitable horseradish peroxidase-conjugated secondary antibodies. The resultant antigen-antibody complexes on membranes were detected by the Western Lightning chemiluminescence reagent (no. NEL105001EA; PerkinElmer).

### Confocal fluorescence microscopic analysis

The procedures for immunofluorescence staining of cell cultures have been described previously (46, 47). Briefly, 293T or HH-B2 cells grown on coverslips were transiently transfected with the indicated expression plasmids for 24 h. Cells were fixed with 4% paraformaldehyde in phosphate-buffered saline (PBS) at room temperature for 12 min and then permeabilized with 0.1% Triton X-100 in PBS for 12 min at room temperature. After incubation with blocking solution (CAS-Block; Invitrogen) for 30 min, cells were exposed to the primary antibody for 1 h. Specific primary antibodies against the FLAG tag (F1804 or F7425; Sigma), γH2AX (9718S; Cell Signaling), 53BP1 (ab175933; Abcam), RPA 32 kDa subunit (sc-271578; Santa Cruz), GAL4 (sc-510; Santa Cruz), and VP16 (sc-7545; Santa Cruz) were used in the study. The anti-ORF50 antibody was generated in our laboratory (48). After washing with PBS, cells were incubated with appropriate secondary antibodies including goat anti-mouse IgG1/Alexa Fluor 546 (A21123; Invitrogen), goat anti-mouse IgG2a/Alexa Fluor 647 (A21241; Invitrogen), goat anti-rabbit IgG/Alexa Fluor 594 (A11012; Invitrogen), and goat anti-mouse IgG/Alexa Fluor 488 (A11005; Invitrogen). Staining with 4′-6-diamidino-2-phenylindole (DAPI) was performed at room temperature for 15 min. The slides were mounted, and cells were analyzed using a confocal laser scanning microscope (Leica TCS-SP5II). The confocal images were acquired using LAS AF Lite software (Leica) under the same settings. To analyze the degree of the colocalization between target proteins, the Pearson’s correlation coefficient was measured using the the plugin Coloc2 (version 2.1.0) of the Image J software (version 1.52 p, National Institutes of Health, Bethesda, MD). Pearson’s correlation analysis is a digital imaging technique that can estimate the overlapping pixels between two fluorescence signals (or two channels) in microscopy images. Prior to running the Coloc2 in Image J, the regions of interest (ROIs) in the merge images of confocal microscopy were outlined and selected using the “Freehand” selection tool. Pearson’s coefficient values (above threshold) were calculated after running the Coloc2 plugin. The values of Pearson’s correlation coefficients range from −1 to 1. Generally, the Pearson’s coefficient value above 0.5 indicates a moderate to strong colocalization. However, if the value is between 0.1 and 0.5, there is only a weak colocalization. If the value is less than 0.1, there is no apparent colocalization.

### Coimmunoprecipitation

Coimmunoprecipitation assays were carried out as mentioned previously (49). Briefly, 293T cells were cotransfected with the plasmids expressing GFP fusion proteins and the plasmids expressing FLAG-tagged proteins. At 24 h after transfection, cells were harvested and lysed in the coimmunoprecipitation assay buffer (50 mM Tris-HCl pH 7.6, 150 mM NaCl, 1 mM EDTA, 1% Triton X-100, and 1 mM phenylmethylsulfonyl fluoride). Protein lysates were subsequently mixed with GFP-Trap MA beads (no. gtma-100; ChromoTek). After immunoprecipitation, the resultant immune complexes were subjected to Western blot analysis using specific antibodies.

### Luciferase assays

293T cells were seeded on 24-well plates (7 × 10^5^ cells/well) one day before transfection. Each transfection used a ﬁxed amount (0.8 µg) of plasmid DNA including the reporter plasmid pGal4(5×)-Luc and effector plasmids. At 24 h post-transfection, luciferase reporter activities of transfected cell samples were measured using the luciferase reporter assay system (no. E1501; Promega). Fold activation was calculated as the luciferase activity of the reporter construct in the presence of effectors divided by that in the presence of the empty control vector. All experiments were performed at least three times with duplicate samples.

### Statistical analysis

All data were presented as means ± standard deviation (SD). The Mann-Whitney *U* test was used to evaluate differences between samples. All statistical analyses were conducted using SPSS (Statistical Package for the Social Sciences) software (version 18.0; IBM Corporation, Armonk, NY), and the *P*-values less than 0.05 were considered statistically significant.

## SUPPLEMENTAL MATERIAL

Supplemental figures spectrum.02254-23-s0001.pdf Fig. S1 to S4.

ASM does not own the copyrights to Supplemental Material that may be linked to, or accessed through, an article. The authors have granted ASM a non-exclusive, world-wide license to publish the Supplemental Material files. Please contact the corresponding author directly for reuse.
