# Supplementary material for: Interaction and assembly of the DNA replication core proteins of Kaposi’s sarcoma-associated herpesvirus
Source: Microbiol Spectr. 2023 Oct 24;11(6):e02254-23. doi: 10.1128/spectrum.02254-23 (PMC10715029; doi:10.1128/spectrum.02254-23)
Supplement: Supplemental figures — Fig. S1 to S4. [file spectrum.02254-23-s0001.pdf]

SUPPLEMENTAL INFORMATION

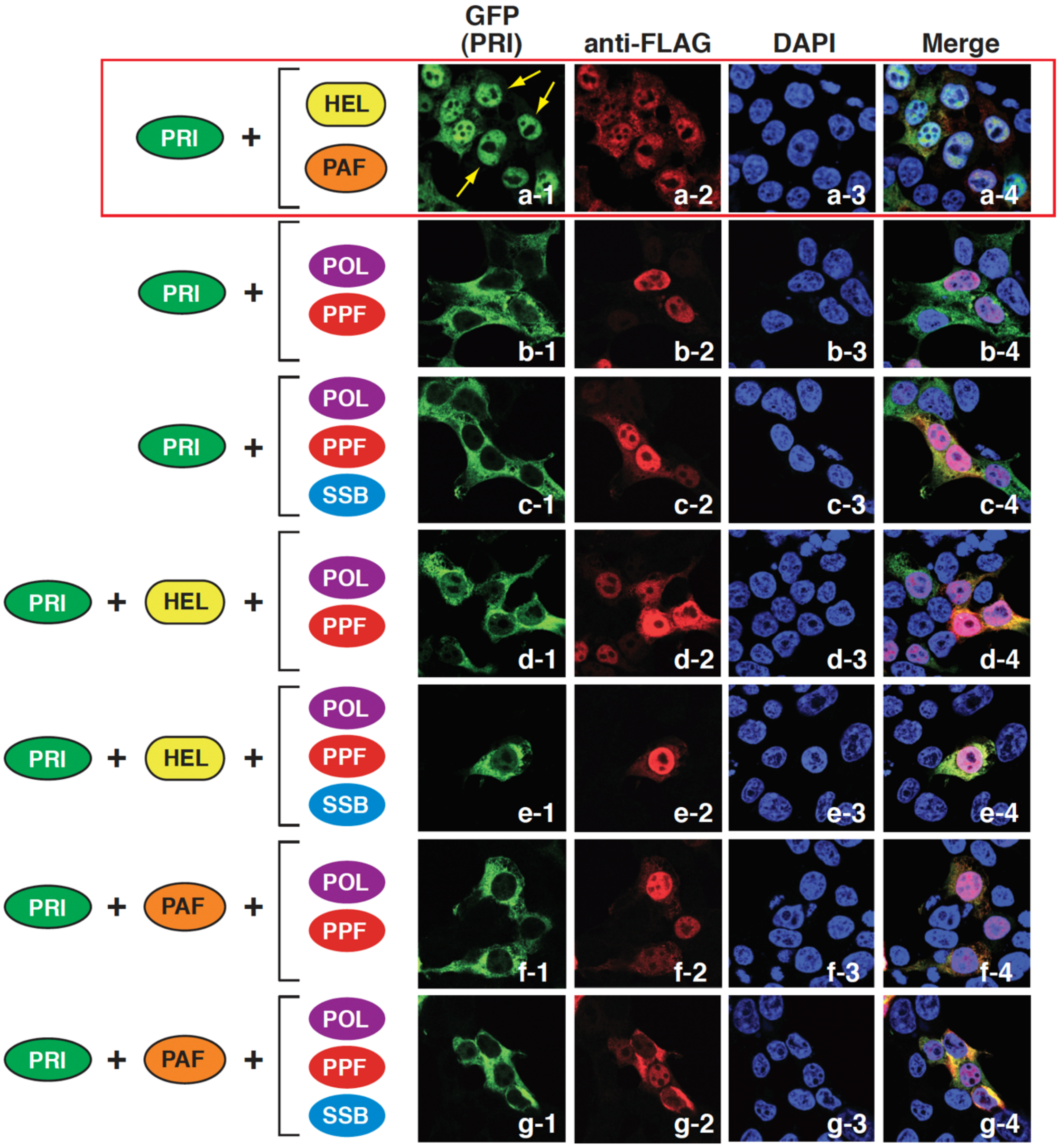

Supplementary Fig. S1. Both ORF44 and ORF40/41 are required for the nuclear transport of ORF56. The plasmid encoding GFP-ORF56 (PRI) was cotransfected with different cominations of the plasmids encoding F-ORF44 (HEL), F-ORF40/41 (PAF), F-ORF9 (POL), F-ORF59 (PPF) and F-ORF6 (SSB) into 293T cells. The subcellular localization of GFP-ORF56 and FLAG-tagged core replication proteins was analyzed by confocal fluorescence microscopy. The nuclear transport of GFP-ORF56 (PRI) mediated by F-ORF44 (HEL) and F-ORF40/41 (PAF) is highlighted in red box. All experiments were repeated at least three times independently with similar results.

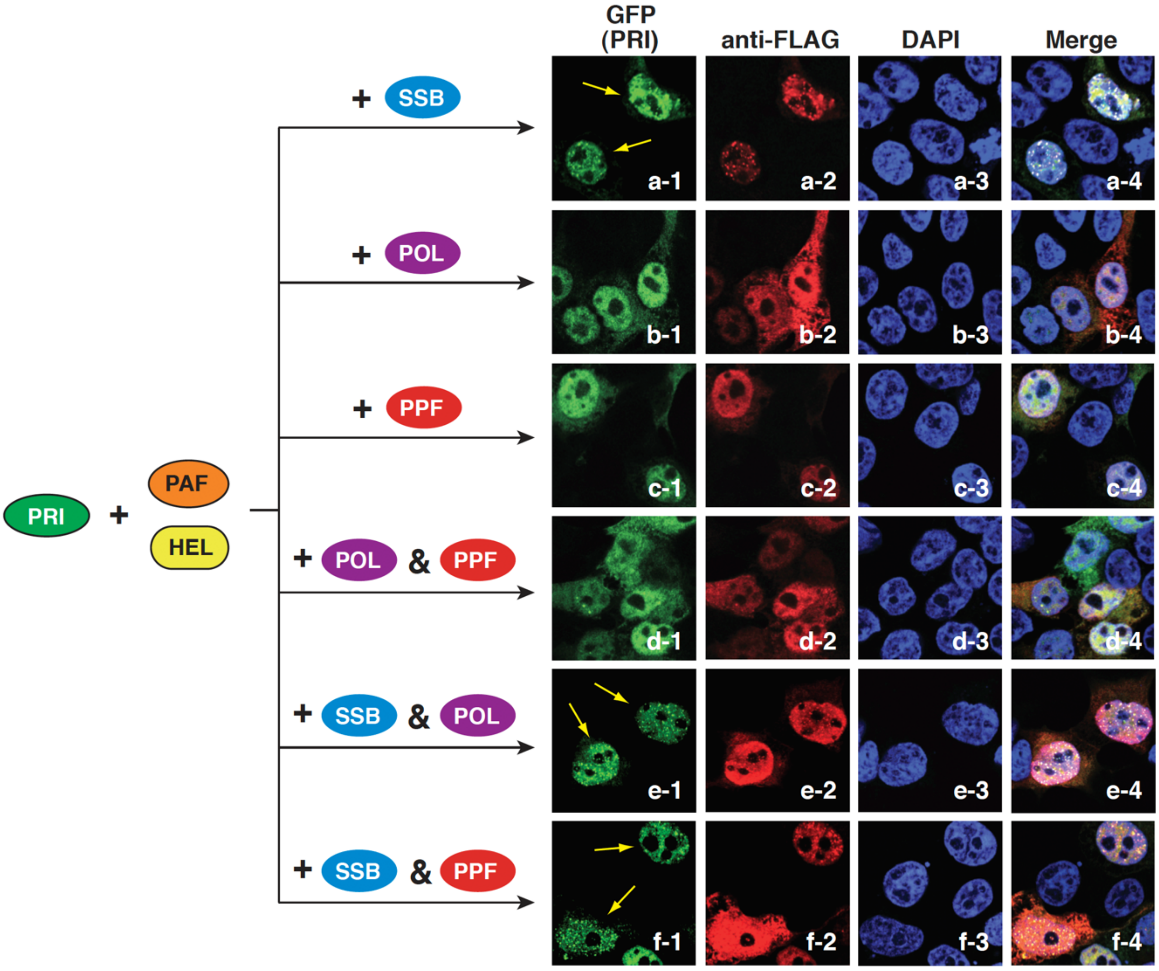

Supplementary Fig. S2. ORF6 (SSB) plays a key factor in promoting the formation of ORF56 puncta in the nucelus. After coexpression of the trimeric helicase-primase subcomplex containing GFP-ORF56 (PRI), F-ORF44 (HEL) and F-ORF40/41 (PAF) with other core replication proteins including F-ORF6 (SSB), F-ORF9 (POL) and F-ORF59 (PPF) in 293T cells for 24 h, the subcellular localization of GFP-ORF56 and FLAG-tagged core replication proteins was analyzed by confocal fluorescence microscopy. Yellow arrows indicate cells with GFP-ORF56 puncta. All experiments were performed at least three times independently with similar results.

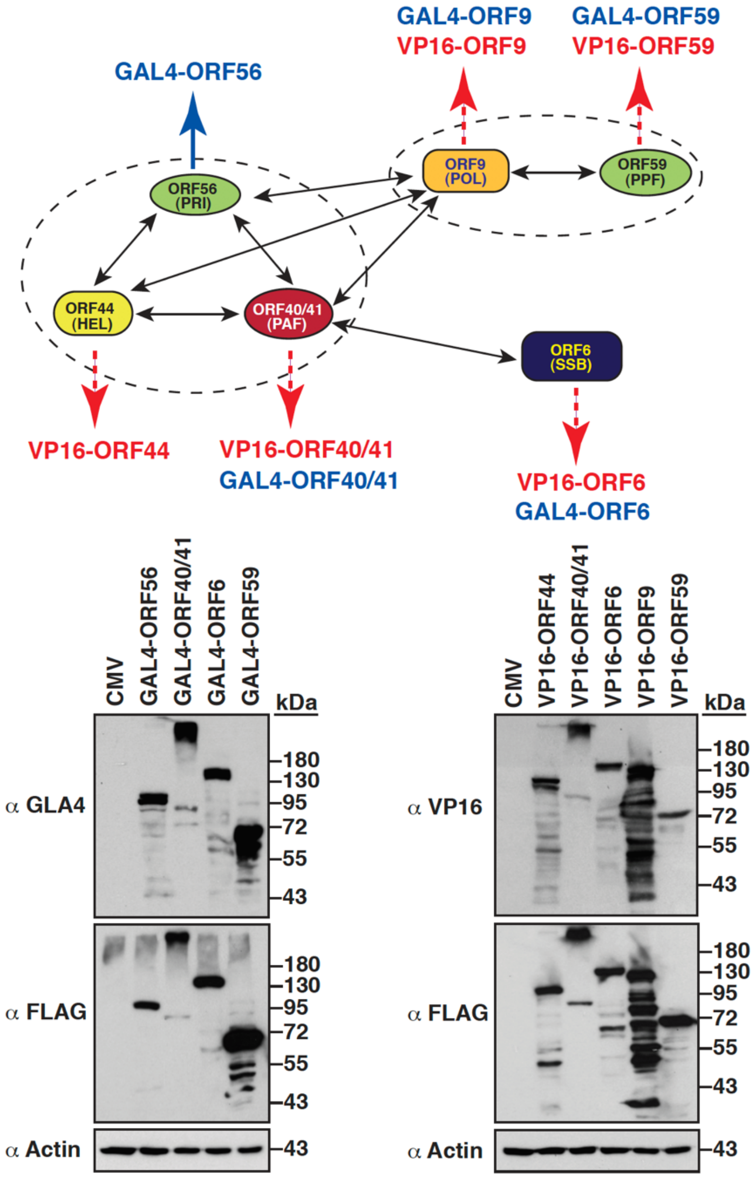

Supplementary Fig. S3. Representative Western blot analysis of GAL4- and VP16-fusion proteins expressed in 293T cells. After 293T cells were transfected with the indicate expression plasmids for 24 h, transfected cells were harvested and subjected to Western blot analysis using antibodies against GAL4, VP16 or the FLAG tag. The experiments were independently repeated twice with similar results.

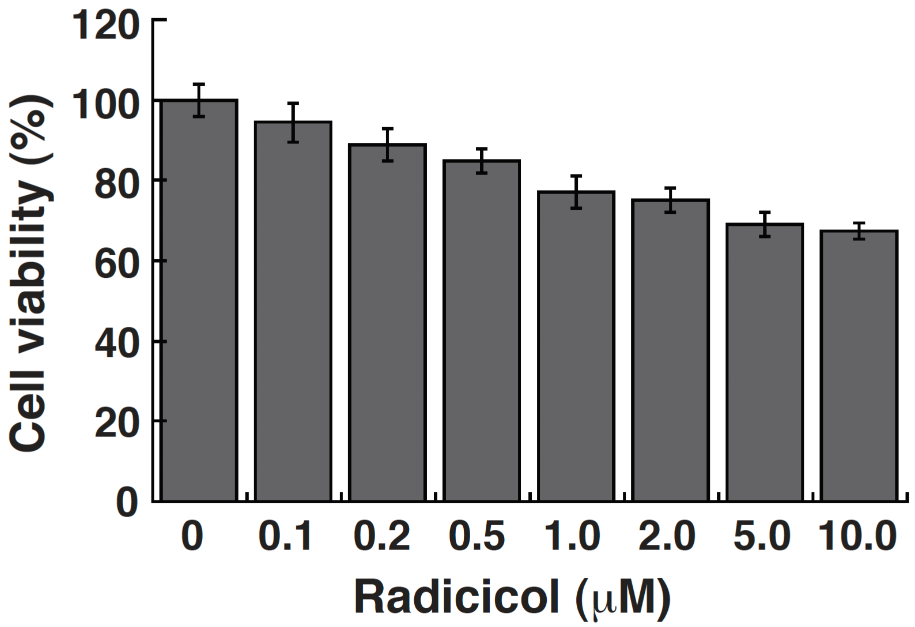

Supplementary Fig. S4. Effect of radicicol on cell viability of 293T cells. After teatment of 293T cells with different concentrations of radicicol for 24 h, the cell viability was measured by Cell Counting kit-8 (CCK-8; #96992, Sigma-Aldrich). Data were presented as mean ± standard deviation (SD) (n = 3).
